# *AcNAC10*, regulated by AcTGA07, enhances kiwifruit resistance to *Pseudomonas syringae* pv. *actinidiae* via inhibiting jasmonic acid pathway

**DOI:** 10.1186/s43897-024-00143-x

**Published:** 2025-04-04

**Authors:** Chao Zhao, Wei Liu, Chenxiao Yao, Yali Zhang, Xiaofei Du, Chao Ma, Rui Li, Hua Wang, Lili Huang

**Affiliations:** State Key Laboratory for Crop Stress Resistance and High-Efficiency Production and College of Plant Protection, Northwest A&F University, Yangling, 712100 People’s Republic of China

**Keywords:** *AcNAC10*, Jasmonic acid, Kiwifruit canker, *Pseudomonas syringae* pv. *actinidiae*, Transcriptional cascade

## Abstract

Kiwifruit bacterial canker is a devastating disease caused by *Pseudomonas syringae* pv. *actinidiae* (*Psa*). NAC transcription factors play a significant role in host immunity. However, the potential molecular mechanism of resistance to semi-biotrophic *Psa* mediated by NAC transcription factors in kiwifruit remains unclear. In this study, we identified a typical NAC transcription factor, *AcNAC10*, which is involved in the jasmonic acid (JA) pathway and is highly expressed in resistant variety RH12 responsing to *Psa*. By overexpression and silencing of *AcNAC10* in kiwifruit, it plays a positive role in enhancing kiwifruit resistance. Likewise, heterologous expression of *AcNAC10* in transgenic *Arabidopsis* and tomato enhanced resistance to *P. syringae*. By directly binding to the promoter of *AcLOX3*, *AcNAC10* inhibited its expression as a transcriptional suppressor. Using a yeast one-hybrid screening library, electrophoretic mobility shift assay (EMSA), and dual-luciferase reporter assays, it showed that AcTGA07 can activate the expression of *AcNAC10*. Moreover, we demonstrated that *AcTGA07* decreased JA accumulation independently of the *AcNAC10*-*AcLOX3* pathway. Our study elucidated the transcriptional cascade regulatory network of *AcTGA07-AcNAC10-AcLOX3*, which enhanced the disease resistance of kiwifruit to *Psa* by inhibiting JA synthesis.

**Supplementary Information:**

The online version contains supplementary material available at 10.1186/s43897-024-00143-x.

## Core

Jasmonic acid (JA) negatively regulated kiwifruit resistance to* Pseudomonas syringae* pv. *actinidiae* (*Psa*). The NAC transcription factor AcNAC10 improved the resistance of kiwifruit leaves to *Psa* by inhibiting the accumulation of JA content. Further studies showed that AcTGA07 could activate the expression of *AcNAC10* by binding to its promoter. Interestingly, our study elucidated the transcriptional cascade regulatory network of *AcTGA07-AcNAC10-AcLOX3*, which enhanced the disease resistance of kiwifruit to* Psa* by inhibiting JA synthesis.

## Gene & Accession Numbers

Gene sequence information was obtained from the kiwifruit database (http://kiwifruitgenome.org/). The accession numbers of the genes used in this study are as follows: *AcNAC10* (Actinidia12297), *AcLOX3 *(Actinidia26023),* AcTGA07* (Actinidia17695), *AcPDF1.2* (Actinidia27667), *AcPDF1.1* (Actinidia25332), *AcAOC1* (Actinidia01662), *AcAOC2* (Actinidia15866), *AcAOC4* (Actinidia18123), *AcLOX1* (Actinidia25766), *AcLOX2* (Actinidia16290), *AcLOX4* (Actinidia40183), *AcLOX5* (Actinidia30006), *AcAOS1* (Actinidia22580), *AcAOS2* (Actinidia15286), *AcAOS3* (Actinidia29848), *AcAOS4* (Actinidia29849), *AcERF1* (Actinidia09555), *AcERF2* (Actinidia02179).

## Introduction

The kiwifruit bacterial canker is the most threatening disease in the kiwifruit industry and is caused by the destructive bacterial pathogen *Pseudomonas syringae* pv. *actinidiae* (*Psa*) (Pinheiro et al., 2020; Lee et al. 2017; Wang et al., 2024). The pathogen can colonize plants for a long time without causing any symptoms until appropriate environmental conditions are available. Systemic infections occur via various pathways (Donati et al. 2020), and *Psa* can infect young kiwifruit twigs systemically within a few minutes (Michelotti et al. 2018). Bacterial canker has spread widely in kiwifruit cultivation areas, owing to the strong adaptability and infectivity of the pathogen. In recent years, the disease has received increasing attention in major kiwifruit planting areas owing to great economic losses (Vanneste 2017; Williams et al. 2020; Kim et al. 2019; Chen et al. 2018). Therefore, identifying genes that confer resistance to bacterial canker is important for molecular breeding and disease control.

Most studies suggest that jasmonic acid (JA) is primarily involved in responses to wounding, insect infestations, and necrotrophic plant pathogens (Ghorbel et al. 2021; Macioszek et al. 2023; Zhang et al. 2017), and plays an important role in disease resistance and susceptibility of kiwifruit (Nunes da Silva et al. 2022; Wang et al. 2021). Previous studies have shown that resistant kiwifruit *(Actinidia arguta*) can effectively resist pathogen invasion compared with susceptible delicious kiwifruit (*A. chinensis* var*. deliciosa*). It has also been observed that susceptible delicious kiwifruit accumulates significant amounts of JA, indicating that JA accumulation may mediate kiwifruit susceptibility (Nunes da Silva et al. 2022). Similarly, the susceptible variety, *A. chinensis* var*. chinensis* ‘Hongyang’(HY), accumulates more JA than the resistant variety, *A. chinensis* var*. deliciosa* 'Jinkui', and studies have found that overexpression of *AcMYB16* in 'Jinkui' kiwifruit significantly increases JA accumulation, thereby reducing resistance to the *Psa* pathogen (Wang et al. 2021). Studies have also found that treatment with salicylic acid (SA) significantly enhances the resistance of Hayward kiwifruit plants compared with treatment with methyl jasmonate (MeJA), suggesting that SA may reduce disease susceptibility, whereas MeJA has the opposite effect (Nunes da Silva et al. 2021). Furthermore, in studies of kiwifruit resistance against fungi, it has been found that methyl jasmonate (MeJA) can activate the expression of key JA pathway genes, such as *AcLOX*, *AcAOS*, and *AcAOC*, in kiwifruit, significantly enhancing its resistance to soft rot pathogens (Li et al. 2022). In summary, JA plays a role in the differential resistance of kiwifruit to different pathogens. Therefore, further studies are needed to understand how to inhibit JA-mediated susceptibility to canker disease in kiwifruit and enhance disease resistance.

The NAC (NAM/ATAF/CUC) transcription factor (TF) family is an important group of TFs in plants that have a variety of biological functions, including growth and development, stress response, and plant adaptation to the environment (Nuruzzaman et al. 2013; Buscaill & Rivas 2014; He et al. 2016; Wen et al. 2023; Wang Y et al., 2022). Notably, NAC TFs are essential for plant defenses against diseases. They regulate plant resistance to pathogens by reprogramming the transcription of genes related to plant defense and rapidly changing plant survival strategies (Puranik et al. 2012; Olsen et al. 2005; Bi et al. 2023). Studies have shown that some NAC proteins regulate the JA pathway as transcriptional activators (Zhang et al. 2017; Yoshii et al. 2010). In *Arabidopsis*, the expression of *ANAC019*, *ANAC055*, and *ANAC072* are induced by JA and *P. syringae* pv. *maculicola* strain ES4326 (*Psm* ES4326), which mainly relies on MYC2, is a master regulator of the JA signaling pathway (Zhang et al. 2017). Zhou et al. (2018) reported that silencing of the NAC transcription factor TaNAC6 in wheat reduced resistance to OEStpk-V and NAU9918 (Zhou et al. 2018), indicating a positive role for TaNAC6 in broad-spectrum resistance to *Bgt* (*Blumeria graminis* f. sp. tritici). Through feedback regulation, JA induces TaNAC6 and enhances wheat resistance to *Bgt* (Zhou et al. 2018). Recently, researchers found that RIM1, another NAC transcription factor, is a host factor involved in the proliferation of rice dwarf virus (rDV). According to Yoshii et al. (2010), RIM1 functions as a transcriptional regulator of JA signaling and is degraded via the 26S proteasome-dependent pathway in response to JA treatment. NAC TFs play an important role in plant immunity against biotrophic and hemibiotrophic pathogens (Seo et al. 2010). The mechanism of NAC TFs involved in JA-mediated disease resistance has been characterized in model crops (Bu et al. 2008). However, the regulation of the immune response of kiwifruit to *Psa*, particularly the JA pathway, remains unclear.

Our previous weighted gene co-expression network analysis of resistant (RH12) and susceptible (SH14) kiwifruit materials showed that NAC-like TFs were highly represented and predicted to play an important role in resistance to bacterial canker (Zhao et al. 2023). In this study, we compared transcriptomes and overexpressed kiwifruit leaf discs to identify a NAC member, *AcNAC10,* which is induced by *Psa*. We found that AcTGA07 positively regulated the expression of *AcNAC10*. *AcNAC10* encodes a protein that binds to the promoter of *lipoxygenase3* (*AcLOX3*), inhibits JA biosynthesis, and enhances kiwifruit resistance to *Psa*. Our study highlights the critical role of the "AcTGA07-AcNAC10-AcLOX3" signaling cascade in regulating JA synthesis and plant immune defense. This study provides new insights into the JA-mediated responses of plant immune processes to *Psa*.

## Results

### Differential gene expression analysis of *Psa-*resistant and *Psa*-susceptible kiwifruits reveals key NAC transcription factors that correlate with disease resistance

To identify NAC transcription factors in kiwifruit, we analyzed 94 NAC proteins (*Arabidopsis thaliana*) against the HongYang GenomeV3 (http://kiwifruitgenome.org/, accessed on 07/May/2022). In total, we identified 142 non-redundant putative *NAC* genes, which were categorized into ten subgroups according to phylogenetic analysis (Supplemental Fig. S1). We also checked the orthologs of the identified NAC TFs in different kiwifruit varieties (Red5, Hongyang, and *Actinidia eriantha*) and other plants, such as *Arabidopsis* and grape, which revealed 61 NAC orthologs present in *Arabidopsis* and 105 in grape (Table S1). We also found that homologous *NAC* genes were significantly different among different species (Fig. S2 A and C). Gene ontology (GO) analysis of the 142 NAC TFs revealed that they were highly enriched in groups such as "regulation of primary metabolic process" and "transcription regulator activity" (Fig. S3).

Next, we assessed the selection pressure of NAC TFs by analyzing the distribution of Ka/Ks and Ks. We observed the data for Ka/ks in Fig. S2 D are more concentrated than those shown in Fig. S2 B, indicating that *AcNAC* underwent stronger purification selection (Ka/Ks < 1) during evolution. Notably, four NAC TFs shared by the three kiwifruit varieties experienced positive selection pressure (Ka/Ks > 1), implying that these genes may possess certain beneficial adaptive characteristics and may have increased gene frequency to some extent.

**Fig. 1:**
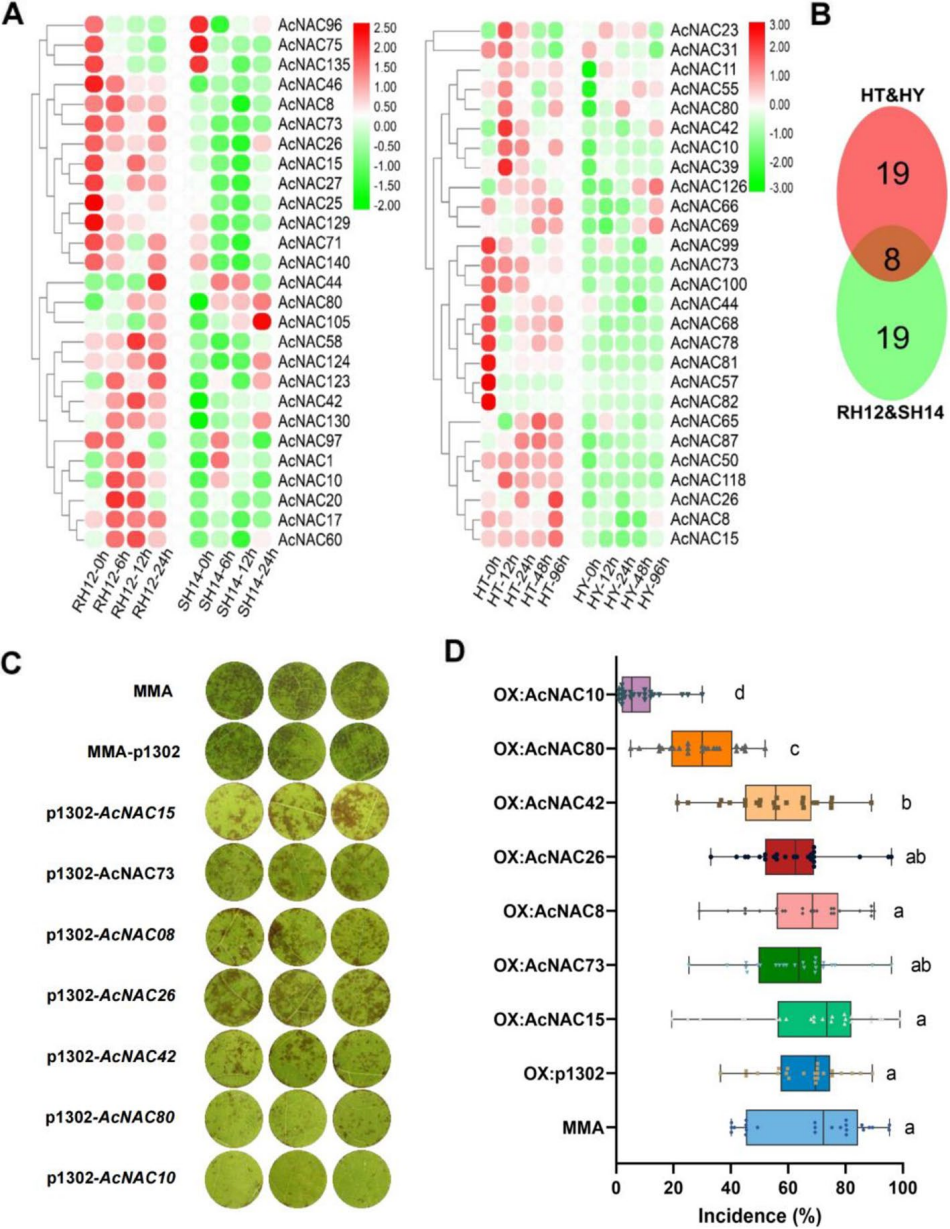
Screening and identification of *AcNAC10* transcription factor in kiwifruit. **A** Heatmap of highly expressed NAC TFs in resistant varieties based on two sets of transcriptome data. Red represents high expression and green represents low expression. HY (susceptibility), *A. chinensis* var. *chinensis* Hongyang; HT (resistance), *Actinidia eriantha* Huate; RH12 (resistance), *A. chinensis* var. *chinensis* RH12; SH14 (susceptibility), *A. chinensis* var. *chinensis* SH14. **B** Calculation of the intersection of NAC TFs that show significantly higher expression in resistant varieties. **C** Identification of disease resistance by overexpression of seven NAC TFs in kiwifruit leaves. p1302, vector p1302-35S-GFP. **D** Statistical analysis of disease incidence. The Least Significant Difference (LSD) test was used for statistical analysis; letters represent significant differences (*p* < 0.05)

Diversity in the evolution of NAC TFs determines their functional differences (Yuan et al. 2019). Therefore, we hypothesized that the differential expression of NAC TFs may correlate with the disease resistance of kiwifruit to *Psa*. To test this hypothesis, we selected two sets of comparative transcriptome data from previous studies using a variety of kiwifruit cultivars (Song et al. 2019). The varieties HY (susceptible) and HT (resistant) belong to *A. chinensis* var. Hongyang and *A. eriantha* Huate, respectively, whereas RH12 (resistant) and SH14 (susceptible) were *A. chinensis* var*. chinensis*. We conducted differential gene expression (DGE) analysis of the two kiwifruit groups (HY vs. HT and RH12 vs. SH14) infected with *Psa* and found 29 NAC TFs (*p* < 0.05) that showed significant DGE in the comparison groups of RH12 & SH14 and HY & HT, respectively (Fig. 1A). When comparing *NAC* expression levels in both comparative transcriptome groups (HY vs. HT and RH12 vs. SH14), we identified eight genes that showed significantly higher expression in resistant varieties than in susceptible varieties (*p* < 0.05) (Fig. 1B). This indicated that the resistance of kiwifruit to bacterial canker disease was correlated with the expression levels of specific NAC TFs. To test this hypothesis, we transiently overexpressed seven *NAC* genes in kiwifruit leaf discs and examined the severity of *Psa* disease (Fig. S4). Interestingly, the overexpression of *AcNAC10* and *AcNAC80* significantly enhanced the resistance of kiwifruit to *Psa* (*p* < 0.05) (Fig. 1C and D). *AcNAC10* is homologous to *NaNAC29* (Table S2) (Ma et al. 2021) and has been reported to be involved in tobacco disease resistance. Therefore, we selected *AcNAC10* as the target gene for further studies.

### AcNAC10 is a nuclear-localized protein that exhibits transcriptional repression

To determine the subcellular localization of AcNAC10, we fused the CDS with green fluorescent protein (GFP) sequence and transiently expressed it in tobacco leaves. The fusion protein showed a predominant nuclear location, indicating that AcNAC10 is a nuclear-localized protein (Fig.S5 A). To determine the transcriptional activity of AcNAC10, we constructed a series of *AcNAC10* truncations. Systematic evolutionary analysis showed that AcNAC10 and NaNAC29 have a close evolutionary relationship, suggesting that they may have functional similarities (Fig. S5 B). Transcriptional activation experiments showed that the *AcNAC10* coding sequence was truncated into three fragments of different lengths (N1, N2, and C), and only the C-terminus grew normally and exhibited blue spots, indicating that full-length AcNAC10 has no transcriptional activation activity and that the transcriptional activation region is located at the C-terminus (Fig. S5 C). There may be an inhibitory domain in the full-length protein to interfere with the function of the C-terminal activation domain.

### *AcNAC10* responds strongly to *Psa* infection and environmental stress

To compare the expression pattern of *AcNAC10* in kiwifruits that are resistant or susceptible *Psa*, we separately inoculated RH12 (resistant) and SH14 (susceptible) with *Psa* and used qRT-PCR to detect the expression in both breeds. We found that at all six testing time points, except 0 hpi (hours post infection), the expression level of *AcNAC10* in the resistant cultivar RH12 was significantly higher than that in the susceptible cultivar SH14, confirming that *AcNAC10* correlated with resistance to *Psa*. (Fig. S5 D). Intriguingly, we found that *AcNAC10* was preferentially expressed in roots and leaves (Fig. S5 E).

To preliminarily speculate on the different biological functions of AcNAC10, we examined the response of AcNAC10 to various external stimuli, such as elf18 (Elongation Factor Tu Receptor 18) induced immune response, H_2_O_2_ oxidative stress, and cold stress. We found that *AcNAC10* expression was greatly elevated by these treatments, indicating that AcNAC10 is involved in multiple stress response processes in kiwifruit (Fig. S5 F).

### Expression of *AcNAC10* is associated with the JA pathway that positively regulates the disease resistance of kiwifruit

**Fig. 2:**
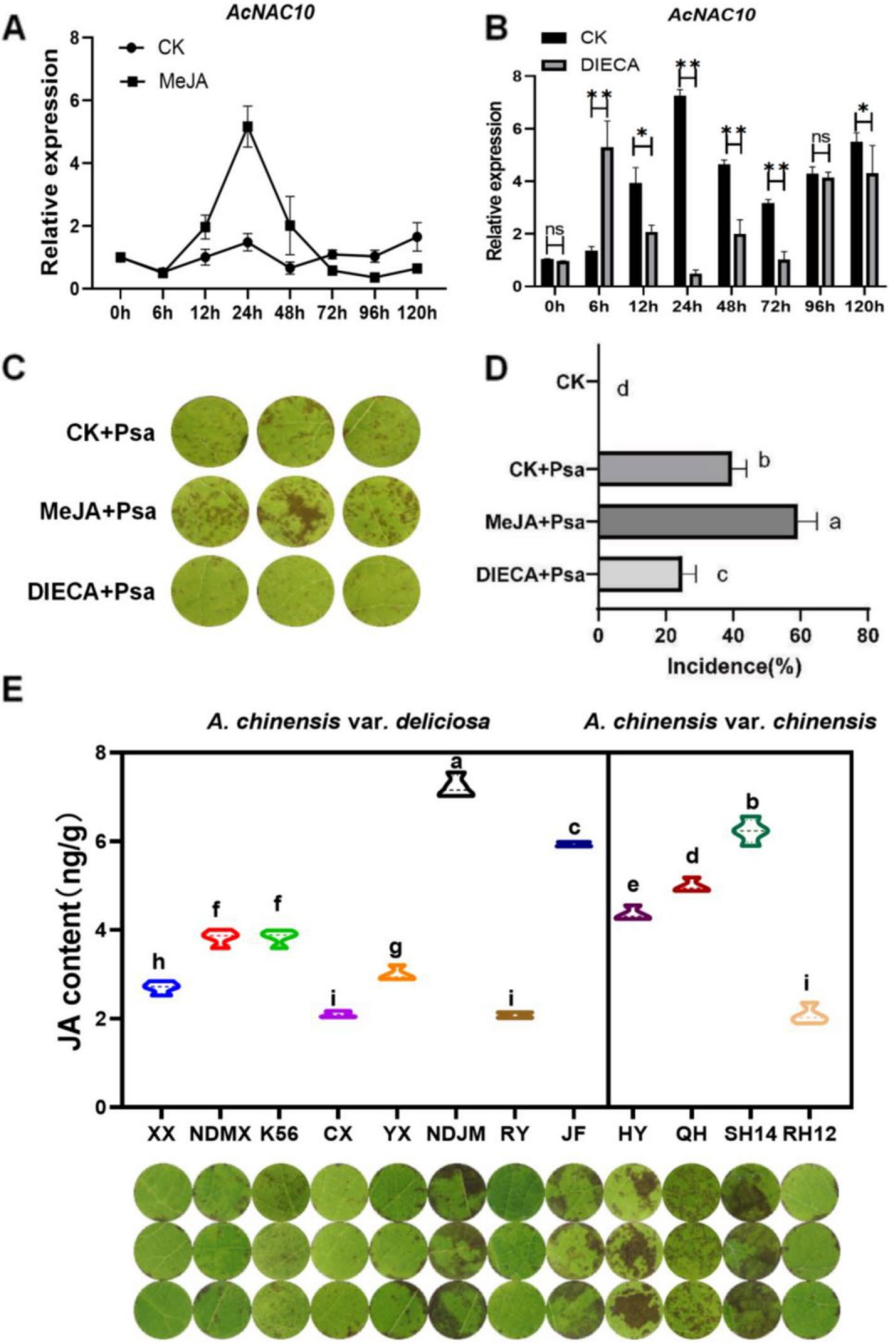
*AcNAC10* is involved in the JA pathway and is associated with disease resistance in kiwifruit. **A** *AcNAC10* expression under MeJA-treated conditions. MeJA, methyl jasmonate. **B** Expression levels of *AcNAC10* in kiwifruit leaves after DIECA treatment. **C** Inoculation of *Psa* after treatment of kiwifruit with DIECA and MeJA. **D** Determination of disease incidence in kiwifruit leaves infected by *Psa*. **E** Testing of disease resistance and JA content in 12 different varieties. NDJM, *A. chinensis* var. *deliciosa* Nongdajinmi; NDMX, *A. chinensis* var. *deliciosa* Nongdamixiang; K56, *A.chinensis* var. *deliciosa* K56; JF, *A. chinensis* var. *deliciosa* Jinfu; YX, *A. chinensis* var. *deliciosa* Yuxiang; RY, *A. chinensis* var. *deliciosa* Ruiyu; CX, *A. chinensis* var. *deliciosa* Cuixiang; XX, *A. chinensis* var. *deliciosa* Xuxiang; HY, *A. chinensis* var. *chinensis* Hongyang; QH, *A. chinensis* var. *chinensis* Qihong. LSD test was used for statistical analysis. Letters represent the significance of differences (*p* < 0.05)

To test whether the JA signaling pathway controls the resistance of kiwifruit to *Psa*, we treated Hongyang leaves with Diethyldithiocarbamate (DIECA), an inhibitor of jasmonate biosynthesis (Yue et al. 2023). Previous studies have reported that the homologous genes, *AcNAC10* and *NaNAC29*, play a role in disease resistance in the JA pathway of tobacco; however, the mechanism of action has not been elucidated (Ma et al. 2021). To examine whether *AcNAC10* is also involved in the JA signaling pathway, we examined the expression of *AcNAC10* in kiwifruits treated with DIECA and MeJA. We found that the expression of *AcNAC10* was inhibited in DIECA-treated samples, whereas it was greatly induced in samples treated with MeJA (Fig. 2A and B). Furthermore, DICEA reduced the colonization ability of *Psa* on kiwifruit, whereas 50 μM MeJA facilitated the colonization of *Psa* in kiwifruit leaves (Fig. S6A), resulting in an increased incidence rate (Fig. 2C and D). This suggests that *AcNAC10* may be involved in the JA signaling pathway.

To further clarify the relationship between *AcNAC10* and the JA pathway, we tested the JA content and *AcNAC10* expression in additional kiwifruit cultivars, including 12 *A. chinensis* cultivars. We found that JA content was positively correlated with incidence in kiwifruit (Fig. 2E) and that the incidence in different kiwifruit cultivars was correlated with JA content (*R* ^*2*^ = 0.7476, *p* < 0.05) (Fig. S6B). Taken together, our results indicate that JA affected the kiwifruit resistance to *Psa*.

### Silencing of *AcNAC10* reduces the resistance of kiwifruit to *Psa*

**Fig. 3:**
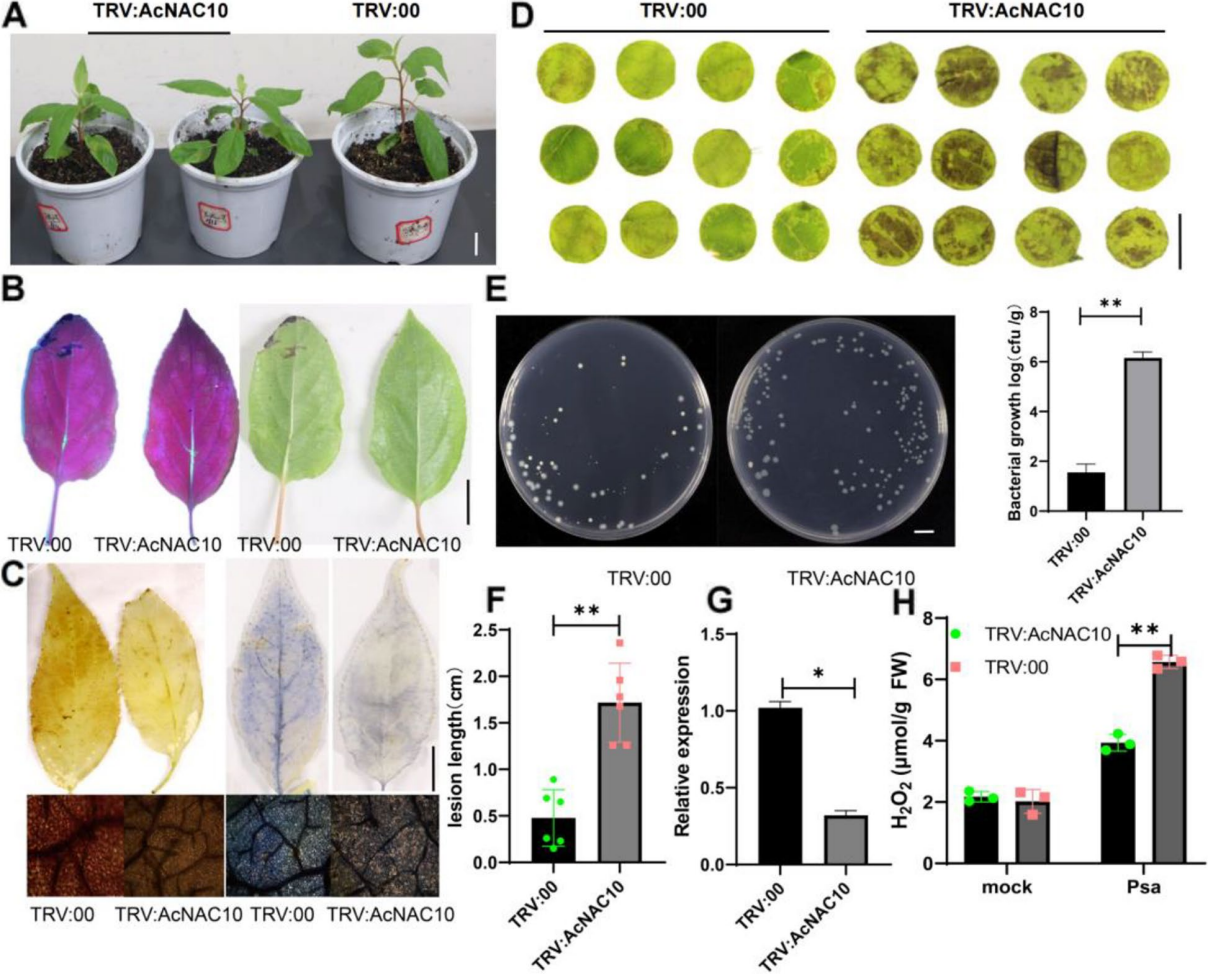
VIGS of *AcNAC10* reduces the resistance of kiwifruit tissue culture seedlings to *Psa* infection. **A** Growth phenotype of kiwifruit after silencing *AcNAC10*. bar = 10 mm. **B** Phenotype statistical analysis (**F**) of kiwifruit leaf veins inoculated with *Psa*. *n* = 6. **C and H** After silencing *AcNAC10* and inoculation with *Psa*, we observed the production of ROS. bar = 1 cm. **D** Detection of disease resistance in kiwifruit by leaf disk assay after silencing of *AcNAC10*. bar = 1 cm. **E** Analysis of the colonization of the pathogen based on the spread plate counting method. **G** Detection of *AcNAC10* expression levels in TRV: *AcNAC10* and TRV:00 silenced plants. Error bars, mean ± standard deviation (SD) of three independent replicates. Asterisks placed above the bars indicate statistical significance, with *p < 0.05 and **p < 0.01, using Student's *t*-test. TRV:00, TRV1 + TRV2; TRV:*AcNAC10*, TRV1 + TRV2-*AcNAC10*. TRV, tobacco rattle virus

NAC TFs play a critical role in regulating plant disease resistance, both positively and negatively (Lee et al. 2017; He et al. 2016; Yokotani et al. 2014). To examine the role of *AcNAC10* in kiwifruit resistance to *Psa*, we silenced this gene in kiwifruit tissue culture seedlings (*A. arguta* var. Longcheng2) using Virus-induced gene silencing (VIGS) method. Silencing of *AcNAC10* produced dwarf kiwifruit seedlings (Fig. 3A and G) and reduced kiwifruit resistance to *Psa* (Fig. 3B, D to F). Simultaneously, the detection of JA content indicated that the silencing of *AcNAC10* promoted JA synthesis (Fig. S7). We also examined the reactive oxygen species (ROS) that plants use to defend against pathogens using diaminobenzidine (DAB) and nitro tetrazolium blue chloride (NBT) staining and by measuring the H_2_O_2_ level. We found that *AcNAC10* silenced seedlings demonstrated reduced ROS levels compared with unsilenced ones (Fig. 3C and H), indicating that *AcNAC10* positively regulates the resistance of kiwifruit against *Psa*.

### Heterologous expression of *AcNAC10* in plants enhances disease resistance to *P. syringae* by inhibiting the JA pathway

**Fig. 4:**
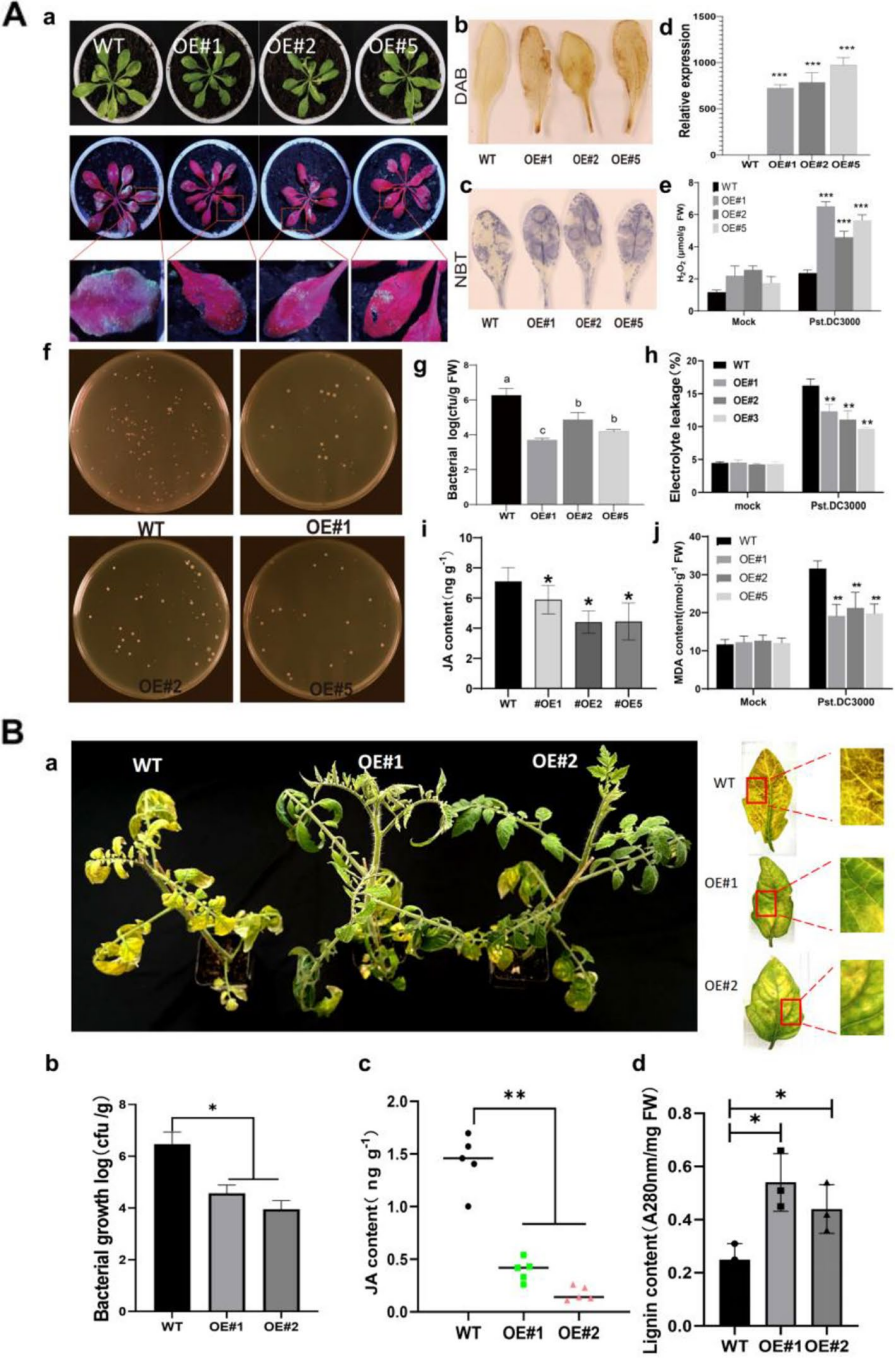
Identification of disease resistance in *Arabidopsis* and tomato stably transformed with kiwifruit *AcNAC10*. **A: a** Phenotypic characteristics of transgenic *Arabidopsis* T2 generation after inoculation with pathogenic bacterium *Pst DC3000*. Green fluorescent protein (GFP) was used to label *Pst DC3000*, which expresses green fluorescence.WT, wild-type; OE, overexpression. **b and c** DAB and NBT staining for reactive oxygen species (ROS) production in tomato after inoculation with *Pst DC3000*. **d** Detection of *AcNAC10* expression in the transgenic *Arabidopsis* plants. **e** Assess the ability of *Arabidopsis* to undergo a production of ROS after pathogen inoculation. **g** Colony counting method for quantifying the bacterial content **(f)** of *Pst DC3000* in *Arabidopsis* leaves. The letters above the bars indicate significant differences according to the LSD test (*p* < 0.05). FW, fresh weight. **h** Electrical conductivity measurements. **i** Detection of JA content. The data shown in the Figure are presented as the mean ± standard error (*n* = 5). **j** Determination of MDA (malondialdehyde) content. The data shown in the Figure are presented as the mean ± standard error (*n* = 3). All experiments were conducted with three biological replicates. Student’s *t*-test was used to determine significant differences between relative expression levels at **, *p* < 0.01.** B: a.** Phenotype of tomato overexpressing after inoculation with *Pst DC3000*. **b** Bacterial colonization density in tomato leaves. **c** Changes in the JA content in tomato plants before and after inoculation with *Pst DC3000*. **d** Lignin content in tomatoes. Asterisks placed above the bars indicate statistical significance, with *p < 0.05 and **p < 0.01, using Student's *t*-test

We first generated transgenic *A. thaliana* that stably overexpressed *AcNAC10* using the floral dip method (Clough and Bent 1998). After screening transgenic seeds of *A. thaliana* using hygromycin (75 mg/ml), we obtained three T2 lines (OE#1, OE#2, and OE#5) that showed resistance to *Pseudomonas syringae* pv. tomato DC3000 (*Pst* DC3000) (Fig. 4A a and d). Pathogen count measurements confirmed reduced colonization of *Pst* DC3000 in the resistance lines (Fig. 4A f and g). Further plant stress tests indicated that compared with the wild-type (WT) line, all three *AcNAC10* lines exhibited increased production of ROS (reactive oxygen species) (Fig. 4A b, c and e) and displayed reduced electrolyte leakage, MDA(Malondialdehyde)content, and JA content (Fig. 4A h, i and j).

In parallel, we generated two stable transgenic tomato lines expressing *AcNAC10*. Similar to observations made in *Arabidopsis*, both tomato *AcNAC10* lines showed resistance to *Pst* DC3000 (Fig. 4B a and b) and stronger plant defense responses, such as elevated ROS, decreased MDA content, and inhibition of excessive chlorophyll degradation (Fig. S9 A to C). We also examined the expression of several JA marker genes, such as *SlAOS3*, *SlMYC2, SlPDF1.2*, and *SlLOX3*, upon *Pst DC3000* infection and found that these genes were downregulated at certain time points (Fig. S9 D to G). Thus, our findings confirm that AcNAC10 enhances the resistance of transgenic *Arabidopsis*.

JA accumulation and lignin deposition often occur during plant immune responses (Zhu et al. 2022). To further explore the influence of *AcNAC10* on JA and lignin contents, we examined the JA content in *AcNAC10* tomato lines using high performance liquid chromatography-tandem mass spectrometry (HPLC–MS/MS). We found that transgenic plants had less JA accumulation than WT plants upon *Pst* DC3000 infection (*p* < 0.05) (Fig. 4B c). However, quantitative detection and safranin-O/fast green staining showed that the AcNAC10 transgenic tomato significantly increased lignin deposition by approximately two times compared with the WT (*p* < 0.05) (Fig. 4B d, Fig. S8). Hence, *AcNAC10* enhanced disease resistance by inhibiting JA in transgenic tomatoes.

### *AcNAC10* directly represses the expression of *AcLOX3* by binding to its NACRS promoter

To address whether AcNAC10 directly regulates the JA signaling pathway, we first searched for key JA marker genes in kiwifruit. Using a homology search of marker genes from model plants, we identified 22 JA marker genes in kiwifruit (Fig. S10. A and B). When examining the expression patterns of these genes, we found four significantly upregulated genes (*AcAOS2*, *AcLOX3*, *AcAOS4*, and *AcLOX1*) in the *AcNAC10*-silenced kiwifruit. Conversely, overexpression of *AcNAC10* resulted in downregulation of these genes (Fig. S10. A). Thus, we speculated that these four genes were directly regulated by *AcNAC10*. Because AcNAC10 is a TF, we tested whether it could bind to the promoters of the four JA markers. The promoter regions of the four genes were amplified using PCR and fused with the GUS reporter gene (pro: genes-GUS). Intriguingly, when promoter/GUS fusions were co-expressed with 35S: *AcNAC10* in *N. benthamiana* leaves, we found that the *AcLOX3* promoter/GUS fusion produced weak GUS activity, indicating that AcNAC10 binds to the *AcLOX3* promoter and inhibits its gene expression (Fig. S10 C). We also examined the expression of *AcLOX3* upon *Psa* infection and found that it showed an opposite trend to that of *AcNAC10* (Fig. S10 D).

**Fig. 5:**
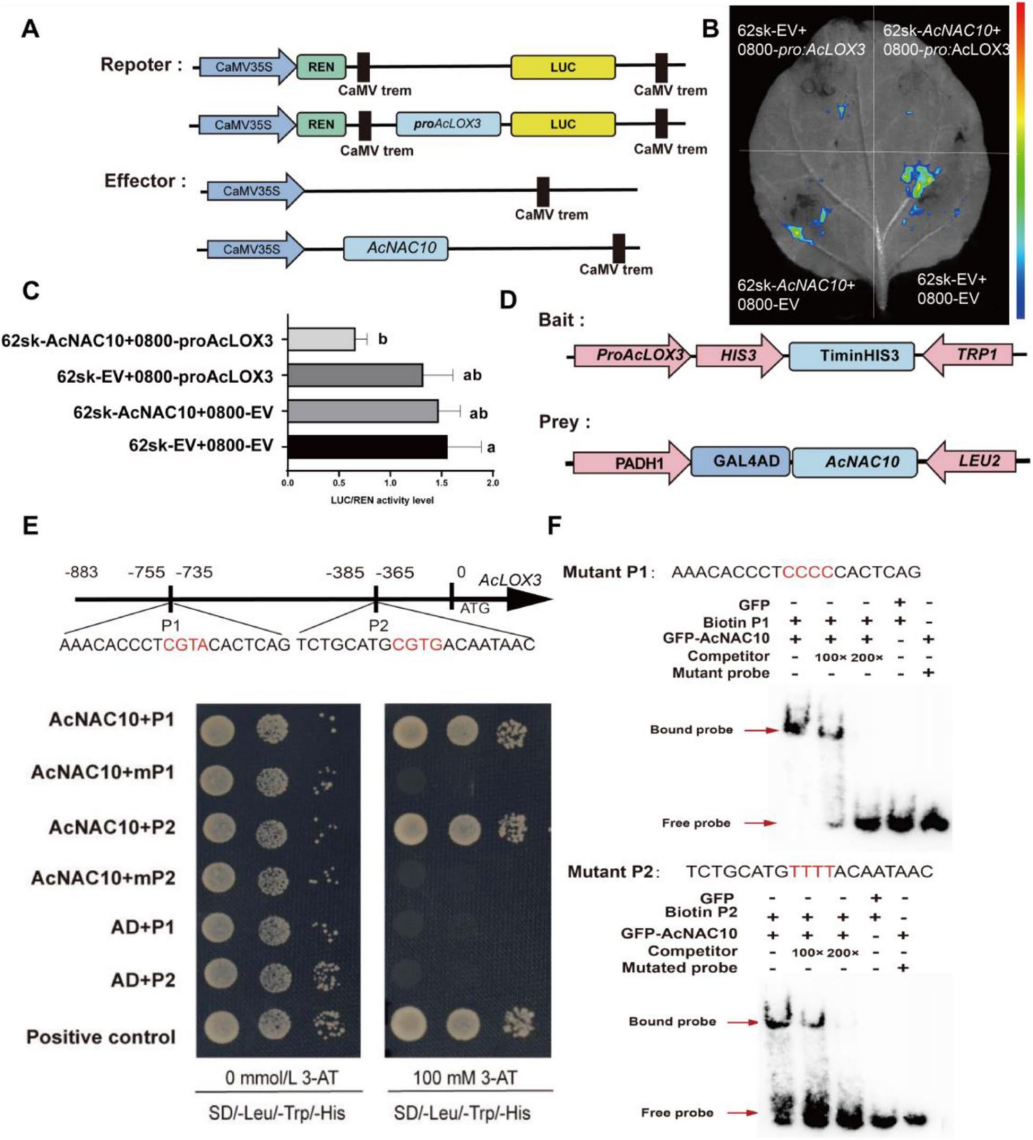
*AcNAC10* binds to the *AcLOX3* promoter and suppresses *AcLOX3* expression. **A** and **B** Luciferase assays in tobacco leaves. p62SK-EV, p62SK-empty vecto; p0800-EV, pGreenII0800-empty vectors. Student’s *t*-test determined significant differences between relative expression levels at ** *p* < 0.01. **C** The ratio of dual luciferase reporter LUC/REN. **D** Schematic diagram of the yeast one-hybrid vector construction using pHis2 and pGADT7. AD and pHis-p53 (positive control) in Y187 cells. The AD-AcNAC10 (pGADT7-*AcNAC10*) and pHi2-pro*AcLOX3* combination in Y187 cells was used as the experimental group. The pGADT7 and pHis2-pro*AcLOX3* were used as the controls. **E** Schematic diagram of the *AcLOX3* promoter structure and Yeast one-hybrid assay validating that *AcNAC10* can interact with the NACRS site of the *AcLOX3* promoter. **F.** Electrophoretic mobility shift assay (EMSA) validated that *AcNAC10* can bind to the NACRS site

To verify whether *AcLOX3* was a direct target of *AcNAC10*, we performed a Dual-luciferase assay. *AcNAC10* significantly inhibited the expression of the LUC reporter gene (*p* < 0.01), which was consistent with the GUS promoter activity analysis (Fig. 5 A and B). In addition, yeast one-hybrid experiments suggested that *AcNAC10* interacts with the *AcLOX3* promoter (Fig. S11).

**Fig. 6:**
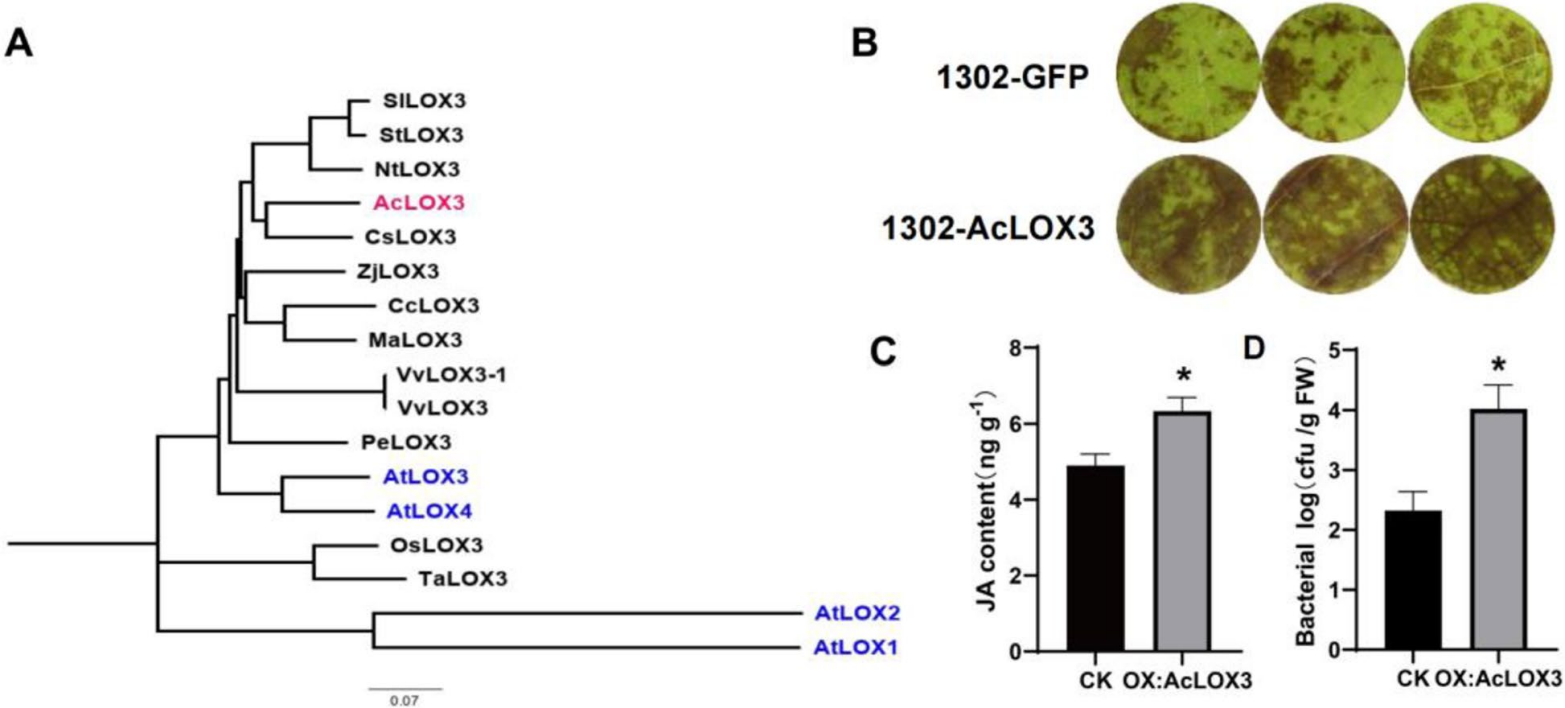
Evolutionary analysis and functional identification of AcLOX3. **A** Evolutionary analysis of homologous protein sequences of AcLOX3. CsLOX3 (*Citrus sinensis*, KAH9713635.1); CcLOX3 (*Corymbia citriodora* subsp. *variegata*, KAF8011508.1); MaLOX3 (*Malus domestica*, RXH88866.1); NtLOX3 (*Nicotiana tabacum*, XP016495606.1); PeLOX3 (*Populus euphratica*, XP011035732.1); SlLOX3 (*Solanum lycopersicum*, AAB65767.1); StLOX3 (*Solanum tuberosum*, KAH0763251.1); VvLOX3 (*Vitis vinifera*, RVW52160.1); VvLOX3-1 (*Vitis vinifera*, NP001290017.1); ZjLOX3 (*Ziziphus jujuba* var. *spinosa*, KAH7547072.1); OsLOX3 (*Oryza sativa*, KAB8090480.1); TaLOX3 (*Triticum aestivum*, XP044371271.1); AtLOX1 (*Arabidopsis thaliana*, AT1G55020); AtLOX2 (*Arabidopsis thaliana*, AT3G45140); AtLOX3(*Arabidopsis thaliana*, AT1G17420); AtLOX4 (*Arabidopsis thaliana*, AT1G72520). **B** Phenotypic observation after overexpression of kiwifruit *AcLOX3*. **C** Detection of JA content after *AcLOX3* overexpression. **D** Colonization levels in bacterial leaf discs. Statistical analysis was performed using the Student's *t-*test. *, *p* < 0.05. The experiment was conducted with at least three biological replicates

To identify the active sites that interact with *AcNAC10*, we employed newly designed primers specific to the promoter region of *AcLOX3* based on Hongyang Genome V3 (Wu et al. 2019). Subsequently, we successfully amplified and cloned an 883 bp promoter sequence. During this process, we identified two previously reported NAC recognition sites (NACRS) (Lindemose et al. 2014; Tran et al. 2004) within the 883 bp promoter region of *AcLOX3* (Fig. S11). Furthermore, Yeast one-hybrid experiments demonstrated that AcNAC10 interacted with the P1 and P2 sites, as indicated by abnormal yeast growth upon mutation of these sites (mP1 and mP2) (Fig. 5E). Additionally, EMSA experiments confirmed the binding of AcNAC10 to biotin-labeled probes for P1 and P2 (Fig. 5F). Systematic evolutionary analysis (Fig. 6A) revealed that AcLOX3 is homologous to LOX3 from *Arabidopsis thaliana*, implicating its role in the regulation of JA content. Consequently, our findings collectively suggest that *AcNAC10* acts as a transcriptional activator that directly regulates the promoter of the JA marker *AcLOX3* by interacting with these two NAC sites.

The LOX gene in plants encodes lipoxygenase, an enzyme that serves as a key enzyme in JA synthesis. Expression of the *LOX* gene promotes the accumulation of JA (Grimes et al. 1992). Therefore, we conducted a systematic evolutionary analysis of the homologous protein *AcLOX3* and found it to be closely related to *AtLOX3* (Fig. 6A), which is one of the four *LOX* genes in *Arabidopsis thaliana*. Studies have indicated that the expression of *LOX3* is inhibited, resulting in blocked synthesis of JAs and defense-related substances (Halitschke et al., 2003; Li et al. 2004). To validate this hypothesis, we overexpressed the *AcLOX3* gene in kiwifruit leaf discs and observed that AcLOX3 promoted the biosynthesis of JA while reducing disease resistance, leading to pathogen colonization (Fig. 6B to D).

### AcNAC10 is positively regulated by the TGA (TGACG SEQUENCE-SPECIFIC BINDING PROTEIN) transcription factor *AcTGA07*

**Fig. 7:**
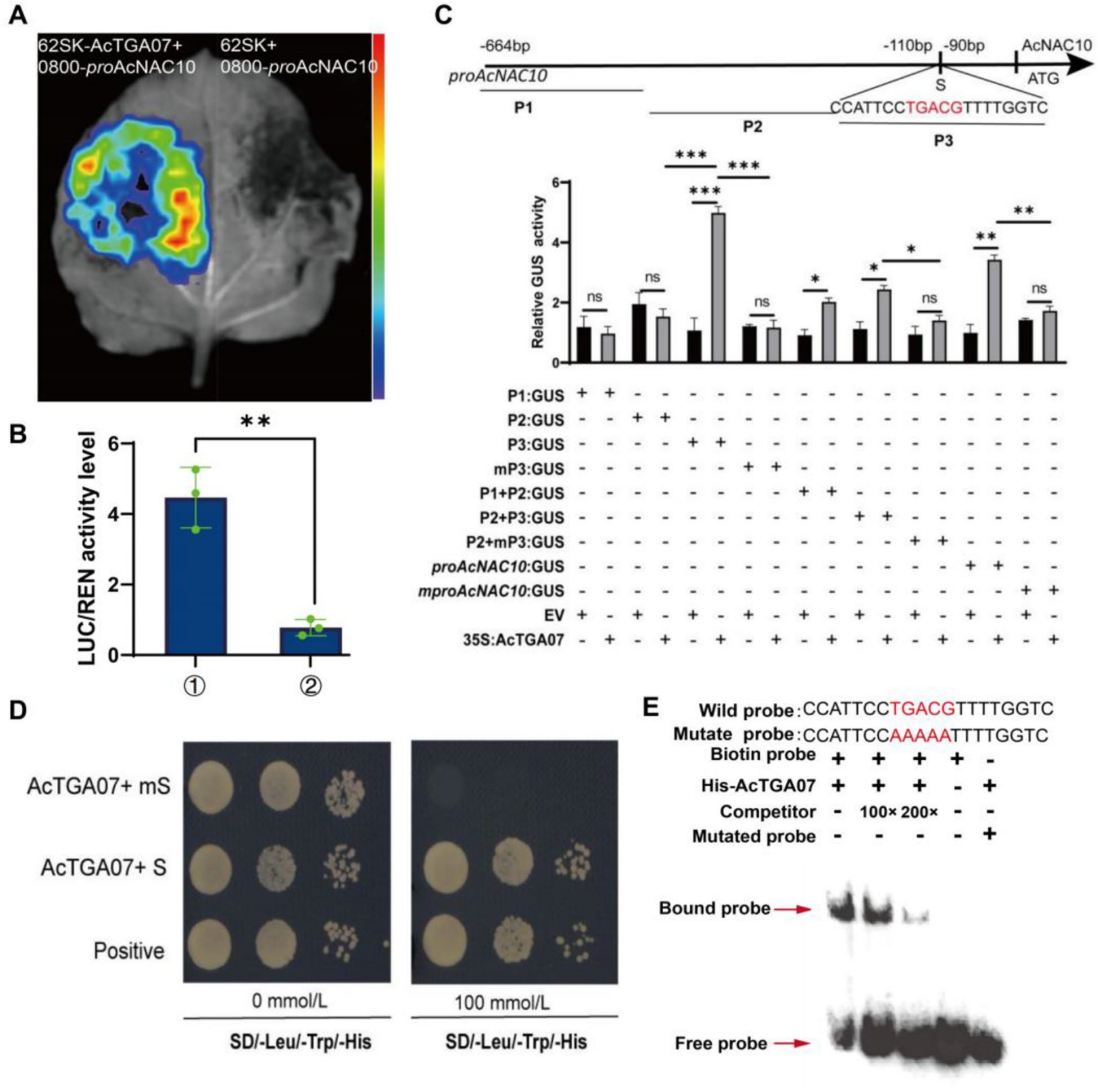
AcTGA07 promotes *AcNAC10* expression by directly binding to the JA-responsive element (TGACG-motif) of the *AcNAC10* promoter. **A.** Dual luciferase assay confirmed the activating effect of AcTGA07 on the AcNAC10 promoter. **B.** Quantitative analysis was performed to measure the LUC/REN ratio. ①, 62SK-AGTGA07 + 0800-proAcNAC10. ②, 62SK + 0800-proAcNAC10. **C.** GUS activity was used to analyze the activation effect of AcTGA07 on the *AcNAC10* promoter region. The statistical significance of the results was determined using Student's *t*-test, where "ns" indicates not significant, *, *p* < 0.05; **, *p* < 0.01 and ***, *p* < 0.001. Values represent mean ± SD of three technical replicates. **D.** Yeast one-hybrid (Y1H) assay confirmed the ability of AcTGA07 to bind to the TGACG-motif. **E.** EMSA demonstrated that AcTGA07 binds directly to the TGACG motif in the AcNAC10 promoter

To further explore the function of AcNAC10, we cloned its promoter and identified multiple potential transcription factor-binding regulatory sites (Fig. S12A) and an element that responds to the JA hormone within the promoter (Table S3). Furthermore, we constructed the *AcNAC10* promoter region in the pHIS2 vector and screened a kiwifruit cDNA library using the yeast one-hybrid method, identifying 43 potential proteins that may act on the *AcNAC10* promoter, including six TFs (Table S4). To clarify whether the six transcription factors regulate the expression of *AcNAC10*, transcriptome analysis revealed that the expression characteristics of *AcNAC10* were similar to those of *AcTGA07* and *AcTGA06* (Fig. S12B). Therefore, we speculated that *AcNAC10* may be regulated by these three genes. *AcTGA07* and *AcTGA06* have close evolutionary relationships with *TGA2/5/6* genes in *Arabidopsis thaliana* (Table S5, Fig. S12C and E), which are known to be involved in the JA pathway (Zander et al. 2010). Subsequently, we conducted GUS experiments to test the regulatory effect of AcTGA06/07 (Liu et al. 2022) on the activity of the *AcNAC10* promoter and found that AcTGA07 activated the expression of the *AcNAC10* promoter, whereas AcTGA06 had no effect (Fig. S12D), indicating that AcTGA07 activated the potential factor for *AcNAC10* expression. We also performed a dual luciferase assay to confirm the interaction between AcTGA07 and the *AcNAC10* promoter sequence, which showed that *AcNAC10* expression was significantly activated by AcTGA07 (Fig. 7A and B).

The *as1*-like sequences are recognized by basic/leucine zipper (bZIP) TFs of the TGA family (Fode et al. 2008). To determine the role of the *as* −1 site in *AcTGA07*-*AcNAC10* interaction, we performed a mutagenesis analysis of the *AcNAC10* promoter. We found that the 3’-terminus of the *AcNAC10* promoter (P3 fragment in Fig. 7C) is essential for the interaction and that the TGACG motif is critical for activation activity. Yeast one-hybrid assay (Fig. 7D) and EMSA (Fig. 7E) confirmed that *AcTGA07* binds to the TGACG motif of the *AcNAC10* promoter. In summary, our findings suggest that the positive regulation of *AcTGA07* by *AcNAC10* depends on the JA regulatory element TGACG motif.

### *AcTGA07* inhibits JA synthesis independent of the *AcNAC10*-*AcLOX3* pathway

**Fig. 8:**
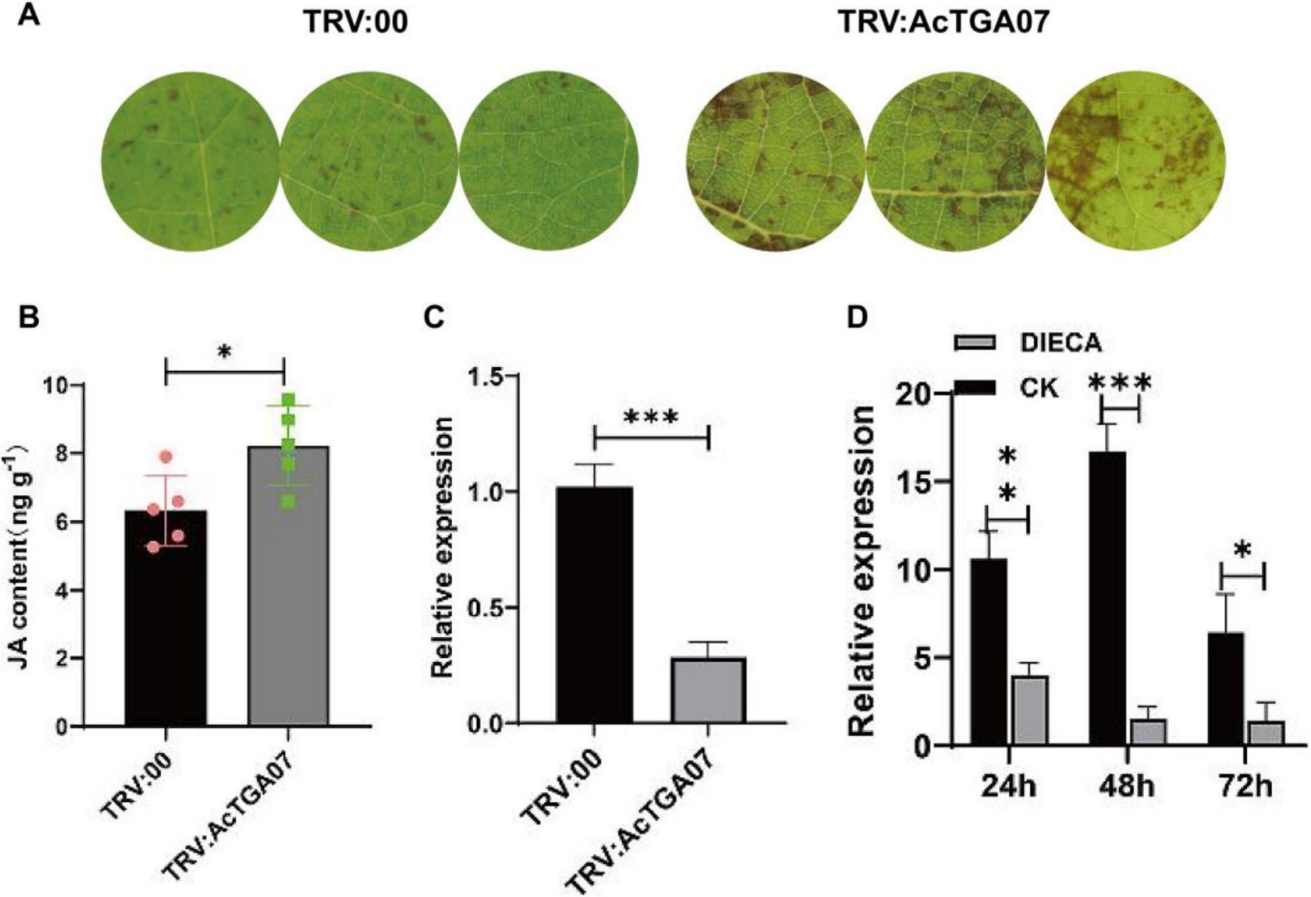
Functional analysis of AcTGA07 in kiwifruit. **A** Phenotypes of silenced AcTGA07 in Kiwifruit leaves. Phenotypes were observed at 0 and 5 days post-injection. TRV:00, TRV1 + TRV2; TRV:*AcTGA07*, TRV1 + TRV2-*AcTGA07*. TRV1, TRV2, or TRV2-*AcTGA07* strains were mixed in equal proportions (v/v, OD_600_ = 0.5) and co-transformed into detached kiwifruit leaves. **B** Detection of JA content after silencing AcTGA07 in kiwifruit plants. Asterisks placed above the bars indicate statistical significance, with * *p* < 0.05, ** *p* < 0.01, and *** p < 0.001, using the Student's *t*-test. **C** Detection of silencing efficiency of *AcTGA07*. OX:*AcTGA07*, overexpressing p1302-35S-*AcTGA07*; OX: EV, overexpressing empty vector (p1302-35S). The expression level of each gene was standardized to that of the reference gene (actin). The experiment was conducted with at least three biological replicates. **D** DIECA can inhibit the expression of *AcTGA07*. Diethyldithiocarbamate (DIECA), an inhibitor of JA biosynthesis. The cultivar used in the study is the disease-resistant variety of kiwifruit, known as the *Actinidia arguta*

To explore the function of AcTGA07 in JA regulation, we silenced *AcTGA07* in kiwifruit leaves. We found that kiwifruit leaves were more susceptible to disease (Fig. 8A). We also found that in the *AcTGA07* silenced line, the level of JA in kiwifruit leaves was significantly increased (*p* < 0.05) (Fig. 8B) and upregulated marker genes (Fig. S13), indicating that AcTGA07 may be involved in inhibiting JA synthesis and positively regulating kiwifruit resistance to *Psa*. A previous overexpression experiment on *AcTGA07* also confirmed the enhanced resistance of kiwifruit to *Psa* (Liu et al. 2022). The qRT-PCR analysis showed that when *AcTGA07* was silenced (Fig. 8C), the expression of JA marker gene *AcLOX3* was increased by approximately 50% (Fig. S13). We also treated kiwifruit leaves with the JA inhibitor DIECA and found that *AcTGA07* was inhibited at three different time points (Fig. 8D). Therefore, our data suggests that AcNAC10 may be located at the core position of the JA signaling pathway and that the expression of AcTGA07 can downregulate the JA pathway gene *AcLOX3* through AcNAC10, thereby enhancing kiwifruit resistance to *Psa*.

**Fig. 9:**
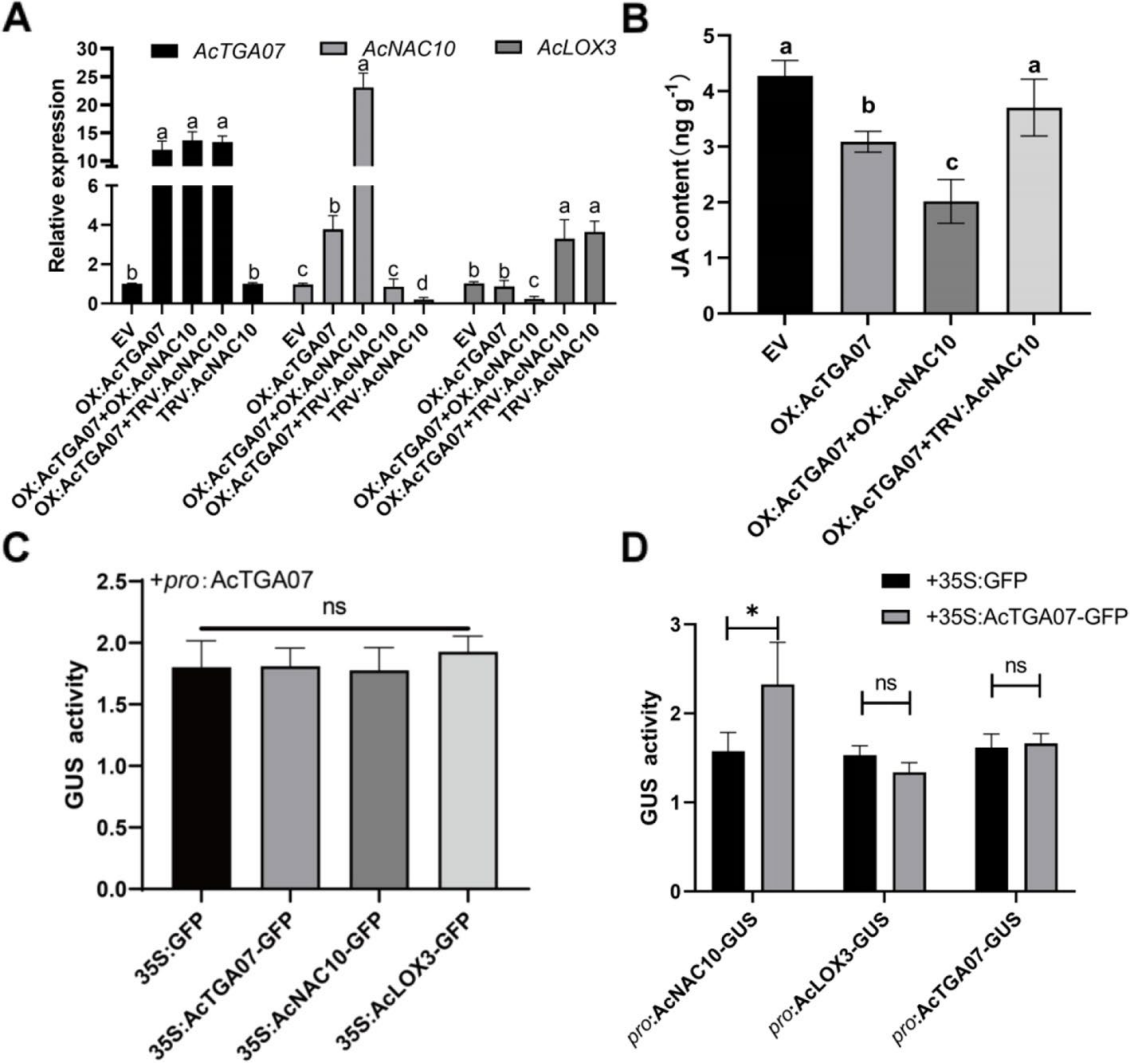
Validation of the regulatory relationship in the “*AcTGA07-AcNAC10-AcLOX3*” cascade. **A** Silencing and overexpressing different genes or gene combinations to detect the relative transcript levels of *AcTGA07*, *AcNAC10*, and *AcLOX3*. Error bars represent the standard deviation of three biological replicates. Different letters above the bars indicate a significant difference (*p* < 0.05) obtained by the LSD test. **B** Changes in JA content. **C** Promoter GUS activity assay to verify the activation effect of 35S:GFP, 35S:*AcTGA07*, 35S:*AcNAC10*, and 35S:*AcLOX3* on the promoter of *AcTGA07*. Statistical analysis were performed using a two-tailed *t*-test. ns, not significant; values are the mean ± SD of three technical replicates (three biological replicates showed similar expression trends, and one was selected for presentation). 35S refers to the strong eukaryotic expression promoter CaMV35S, the fusion expression vector is p1302-GFP, 'pro' is the abbreviation for the gene promoter, and the fusion expression vector is p1381-GUS. **D** Promoter GUS activity assay to verify the activation effect of 35S:*AcTGA07*-GFP on the promoters of JA biosynthesis-related genes *AcLOX3*, *AcNAC10*, and its promoter, *AcTGA07*. LSD test was used for statistical analysis. ns, not significant

To verify whether the regulation of AcTGA07 on downstream JA was completely dependent on AcNAC10, we found that silencing of *AcNAC10* did not affect the expression of *AcTGA07* (Fig. 9A), whereas overexpression of *AcTGA07* significantly enhanced the expression of *AcNAC10*, indicating that *AcTGA07* is upstream of *AcNAC10* and is not regulated by AcNAC10 (Fig. 9A). Furthermore, when both *AcTGA07* was overexpressed and *AcNAC10* was silenced, compared with the control group EV, the expression of *AcLOX3* was inhibited and the JA content was significantly decreased (*p* < 0.05). Similarly, when *AcTGA07* and *AcNAC10* were simultaneously overexpressed, JA accumulation was significantly decreased compared with the overexpression of *AcTGA07* and silencing of *AcNAC10* (*P* < 0.05) (Fig. 9B). This suggests that AcTGA07 inhibited JA synthesis via the *AcNAC10-AcLOX3* pathway (Fig. 9C and D). All results demonstrated that AcTGA07 had no activation effect on the *AcLOX3* promoter, suggesting that AcTGA07 decreased JA content by relying on AcNAC10*.*

## Discussion

The NAC (NAM, ATAF, and CUC) transcription factor superfamily is one of the largest families in the plant kingdom, with members widely participating in the regulation of networks that control many physiological processes including leaf senescence, fruit ripening, growth, development, and stress responses (Ma et al. 2018; Cai et al. 2021; Fan et al. 2018). Emerging evidence suggests that NAC TFs are crucial for the regulation of plant immunity (Yuan et al. 2019; Nuruzzaman et al. 2013; Bian et al. 2020). There is conclusive evidence that NAC TFs act as positive or negative regulators of the immune response of plants against biotrophic or necrotrophic pathogens. In pepper, the interaction between *CaNAC2c* and *CaNAC029* in the nucleus activates JA-mediated immunity and promotes H_2_O_2_ accumulation, thereby enhancing immunity against *Ralstonia solanacearum* infection (Cai et al. 2021). The triple mutation of *anac019*, *anac055*, *and anac072* in *Arabidopsis* enhances the resistance to *P. syringae* pv. *maculicola* ES4326 (Bu et al. 2008). Overexpression of *ANAC042/JUB1* weakened resistance to *Pst DC3000* in *Arabidopsis*, whereas *anac042/jub1* knockdown mutants reduced disease symptoms and colonization by *Pst DC3000*, indicating that *ANAC042/JUB1* inhibits the immunity of *Arabidopsis* to *Pst DC3000* (Wu et al. 2012; Shahnejat-Bushehri et al. 2016). Furthermore, NAC TFs are closely related to JA synthesis regulation during plant disease resistance. In tomato, JA2L, the NAC transcription factor, is activated by JA and COR (*Pseudomonas syringae* pv. tomato produces coronatine). Genetic analysis suggest that JA2-Like (JA2L) mediates the opening of tomato stomata, which is favorable for *Pst DC3000* infection (Du et al. 2014). In rice, the NAC transcription factor *RIM1* participates in defense against the rice dwarf virus (RDV); *rim1* mutants rapidly accumulate massive amounts of endogenous JA when infected, suggesting that *RIM1* may represent a new molecule in JA signaling (Yoshii et al. 2010). Our study revealed that *AcNAC10* is a component of JA signal transduction that plays a role in kiwifruit resistance to *Psa*. There are at least three pieces of evidence that support this. First, *AcNAC10* expression was induced by MeJA and inhibited by DIECA. *AcNAC10* expression was positively correlated with JA synthesis and disease resistance (Fig. 4A and B, Fig. 3). Second, AcNAC10 inhibited the expression of *AcLOX3,* which encodes a lipoxygenase involved in JA synthesis (Fig. 5). Third, the JA-responsive element (*as-1*) of the *AcNAC10* promoter was activated by the transcription factor *AcTGA07* (Fig. 7)*,* which reduced JA synthesis and increased resistance to the simi-biotrophic strain of *Psa*.

Previous reports have shown that jasmonates can antagonize the biotrophic plant defense mediated by SA (Kloek et al. 2001; Kunkel et al., 2002), but there are also reports suggesting that JA and SA can work together to defend against pathogen invasion (Yang et al. 2015; Truman et al. 2007). Our study did not negate the role of SA in kiwifruit disease resistance. Our results support the anti-disease role of JA in hemibiotrophic pathogens. Numerous studies have shown that SA and JA have opposing regulatory effects on kiwifruit (Nunes da Silva et al. 2022; Wang et al. 2021). Our research concluded that JA mediates the susceptibility of kiwifruit to *Psa*. JA-mediated susceptibility is a common phenomenon in the plant kingdom (Gupta et al. 2020), and interactions between *Psa* and kiwifruit are no exception. This suggests that we can draw upon research into other plant resistance mechanisms to study kiwifruit resistance to *Psa*.

Few studies have reported the JA immune regulation mechanism mediated by NAC TFs in kiwifruit, particularly about their resistance to canker disease. *ANAC019* and *ANAC0055*, which are homologous to *AcNAC10*, have been identified in *Arabidopsis thaliana*. Double mutant plants of *anac019* and *anac055* displayed reduced JA-induced expression of VEGETATIVE STORAGE PROTEIN1 (*VSP1*) and LIPOXYGENASE2 (*LOX2*), whereas transgenic plants overexpressing these *NAC* genes exhibited enhanced JA-induced expression (Bu et al. 2008). In our study, AcNAC10 inhibited the *AcLOX3* promoter. Additionally, a functional study of the homologous gene *NeNAC29* in *tobacco* indicated that it plays a role in JA regulation (Ma et al. 2021). These findings suggest that *AcNAC10* represses the defensive response of JA signaling. The molecular biology experiment conducted in this study confirmed that *AcNAC10* restrained the promoter of the first key synthetic enzyme gene, *AcLOX3*, in the JA synthesis reaction. Moreover, it interacts with two reported NACRS sequences (He et al. 2018), participating in the regulation of JA biosynthesis and completing the regulatory cascade. Additionally, the study found that although *AcNAC10* inhibited the synthesis of JA, it promoted the accumulation of lignin (Fig. 3, Fig. S8). These results suggest that *AcNAC10* plays a dual role in the resistance of kiwifruit to disease, particularly in the biosynthesis of lignin and JA.

TGAs have been extensively studied as important regulatory factors in the SA signaling pathway and participate in the immune regulation of plants (Qi et al. 2022; Fonseca et al. 2022). Using the Y1H screening library, we successfully identified an important transcription factor, AcTGA07, which interacts with the *AcNAC10* promoter and activates the expression of *AcNAC10*. AcTGA07, a key player in the JA pathway, is closely related to *TGA2/5/6* in *Arabidopsis* and belongs to the same evolutionary branch, Class II, according to previous classifications of the TGA family (Gatz 2013). Studies have shown that *TGA2/5/6* positively regulates the JA response (Zander et al. 2010). Moreover, studies have demonstrated that the *NPR1* interacts with TGA TFs (such as *TGA2, TGA3, TGA5, and TGA6*) to activate the expression of JA-related genes (Zander et al. 2010; Bargmann et al. 2009). Moreover, a previous study demonstrated that *AcTGA07* is responsive to JA in kiwifruit (Liu et al. 2022). In this study, we confirmed that *AcTGA07* acts as an immune regulatory factor in kiwifruit and negatively regulates JA synthesis (Fig. 8). This finding is significant for advancing our understanding of JA.

Additionally, TGAs can bind to multiple *as* −1 sequences in the SAR (systemic acquired resistance) system for activation (Fode et al. 2008). Our findings suggest that only one of the 13 *TGA* genes, *AcTGA07*, activates the *AcNAC10 promoter*. The reason for the ability of this gene to activate the *AcNAC10* promoter remains unknown. This could be due to differences in TGA protein structure or other unknown factors involved in regulation. We also found that not all promoters carrying *as-1* sequences could be activated by the TGA TFs. Clearly, the interaction between *AcTGAs* and the *AcNAC10* promoter is more complex than previously thought. The cis-acting element composition of the *AcNAC10* promoter in kiwifruit may be different from that in other plant species or may be subject to different regulations. Therefore, the binding of different TGAs to cis-acting element *as-1* in the *AcNAC10* promoter may have varying biological significance.

NAC TFs typically regulate plant physiological characteristics by modulating cascade reactions. For instance, the "*OPpNAC.A59-PpERF.A16-PpACS1*" cascade reaction (*PpNAC.A59*) indirectly regulates ethylene biosynthesis through the NAC-ERF signaling cascade (Guo et al. 2021). In the "GhFSN1-GhMYB7-GhCESA4" cascade reaction, the MYB transcription factor acts as an "intermediate factor," assisting NAC proteins in regulating cellulose synthesis (Huang et al. 2021). We then verified the relationship between JA biosynthetic regulation and the '*AcTGA07*-*AcNAC10*' module. When we co-overexpressed *AcTGA07* and silenced *AcNAC10* in kiwifruit leaves, compared with the simultaneous co-overexpression of *AcTGA07* and *AcNAC10* genes, the JA content in kiwifruit decreased. Therefore, we speculated that *AcTGA07* negatively regulates JA, through the '*AcTGA07*-*AcNAC10*' pathway. Additionally, we also found that MeJA treatment promoted the expression levels of *AcTGA07* and *AcNAC10*, indicating that the '*AcTGA07*-*AcNAC10*' pathway was activated by JA and may function as a negative regulatory chain in the plant body. The mechanism acting as a "brake" mechanism to inhibit excessive JA synthesis still needs further exploration. Cross-regulation may occur through a cascade of reactions involving multiple genes. We used a GUS promoter activity experiment to show that *AcTGA07* did not directly activate the downstream gene *AcLOX3* (Fig. 9). The results depicted in Fig. 9 show that there was no feedback regulation between *AcTGA07, AcNAC10,* and *AcLOX3*. Therefore, we speculate that the TGA-mediated “*AcTGA07*-*AcNAC10*-*AcLOX3*” cascade response is unidirectional.

**Fig. 10:**
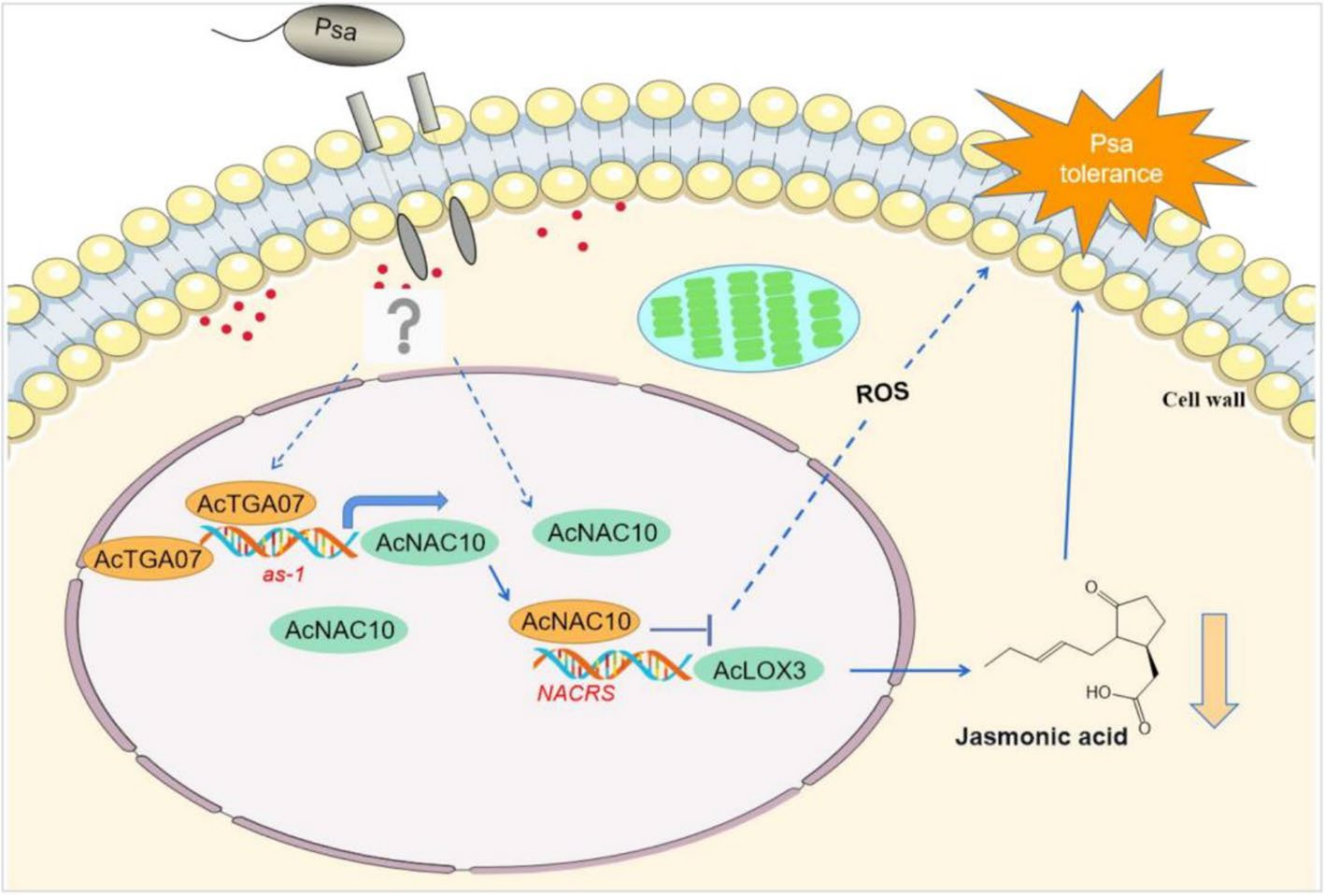
The AcTGA07-AcNAC10-AcLOX3 cascade regulates the network diagram of disease resistance in kiwifruit. ROS, reactive oxygen species; NACRS, NAC recognition site

Based on our findings, we proposed a working model (Fig. 10) in which *AcTGA07*-*AcNAC10*-*AcLOX3* decreased JA accumulation and enhanced kiwifruit resistance to canker disease. In this model, the SA signaling pathway, *AcTGA07,* directly regulated the promoter of *AcNAC10* and negatively regulated JA accumulation under *Psa* stress. Additionally, *AcNAC10* directly binds to NACRS in the *AcLOX3* promoter and suppresses its expression. Reduced AcLOX3 activity results in decreased JA synthesis and a simultaneous increase in ROS levels. Our study revealed how *AcNAC10*-mediated repression of JA synthesis affects the molecular mechanism of kiwifruit resistance to *Psa* and establishes a biological basis for the molecular breeding of kiwifruit for disease resistance.

## Materials and methods

### Plant and bacterial materials

*Actinidia chinensis* varieties RH12, SH14, and Hongyang, which were used for inoculation, were obtained from the greenhouse facilities of the College of Horticulture at Northwest A&F University. *Psa* strain M228 was used for infection tests (Zhao et al. 2019) and the streaked plate was performed on Luria–Bertani (LB) medium (Kan 50 ug/ml). A single colony of *Psa* was selected for further analysis. The culture was shaken for 14 h at 25 °C and centrifuged to enrich bacteria. The concentration of the bacterial suspension was measured using a spectrophotometer and adjusted to OD_600_ = 0.1, resulting in a concentration of approximately 1 × 10^5^ CFU/ml.

### Identification and phylogenetic analysis of kiwifruit *AcNAC* orthologs

To identify the NAC family in kiwifruit, we first selected 94 NAC proteins reported in *A. thaliana* (https://www.Arabidopsis.org/index.jsp, accessed on 07/May/2021) and performed BLASTP searches (E-value < 1e-5) against the *A. chinensis* genome (HongYang.V3, http://kiwifruitgenome.org/, accessed on 07/May/2022). The same searches were performed to identify NAC orthologs in grapes (http://plants.ensembl.org/index.html, accessed on 07/May/2023). Next, Hidden Markov Model (HMM) searches using the NAM (PF02365) domain were conducted to refine orthologs. An evolutionary tree was constructed by the ML (maximum-likelihood) method, where ITOL (https: / / itol.embl.de/, accessed on 01/May/2023) were used to adjust the evolutionary tree.

### GO Annotation of *AcNAC* Gene family synteny and selective pressure analysis

GO analysis was performed using Blast2GO (version 5.0) with the default parameters (Conesa et al. 2005). Full-length amino acid sequences of AcNAC proteins were used as query data and the SwissPort database (https://www.uniprot.org/) served as a reference. GO annotations were categorized into three types: molecular functions, cellular components, and biological processes. The relationship between tandem and fragment repeats in kiwifruit was analyzed using MCScanX(V.2.0) (Wang et al. 2012). Collinear maps among species and varieties were drawn using the Multiple Synteny Plot module in TBtools (V. 2.056) (Chen et al. 2020).

The selection pressure was analyzed by comparing Ka (the number of nonsynonymous substitutions per nonsynonymous site) and Ks (the number of synonymous substitutions per synonymous site) using the KaKs Calculator software (V.2.0) (https://sourceforge.net/projects/kakscalculator2/). A graph was drawn using an R packet (V.4.3.1) (ggplot2).

### Construction of pCAMBIA1302-AcNAC10, TRV2-AcNAC10, pGBKT7-AcNAC10, and pCAMBIA1381-pro: AcLOX3 plasmids

To generate AcNAC10 GFP fusion, AcNAC10 open reading frame (ORF) was PCR-amplified by 2 × Phanta® Flash Master Mix (Dye Plus) (#P520-02, Vazyme, Nanjing) and connected by ClonExpress ® II One Step Cloning Kit (#C112, Vazyme, Nanjing) and cloned into the pCAMBIA1302-35S-GFP plasmid that was digested with Spe I and Nco I.

The 303 bp fragment of the *AcNAC10* gene was amplified by PCR and cloned into the TRV2 vector to construct the fusion expression vector TRV2-AcNAC10. TRV1, TRV2, and TRV2-AcNAC10 were introduced separately into *Agrobacterium* GV3101. Agrobacterium cultures were resuspended in the infiltration buffer.

According to the primers BD-AcNAC10-F/R shown in Table S7, the ORF of AcNAC10 was amplified and purified. The PGBKT7 empty vector was linearized using the restriction enzymes *NdeI an*d *SalI*, and the fusion expression vector BD (PGBKT7)-AcNAC10 was constructed using a ClonExpress ® II One-Step Cloning Kit (#c112, Vazyme, Nanjing).

Specific primers were designed based on the Kiwifruit Genome V3 (http://kiwifruitgenome.org/) to amplify the promoter regions of *AcLOX3*. The purified PCR products of *pro: AcLOX3* were cloned into the linearized pCAMBIA1381-GUS vector to form the fusion expression vector pCAMBIA1381-pro: AcLOX3-GUS. The resulting recombinant vectors were transformed into competent *Agrobacterium tumefaciens* (GV3101) (pSoup19) using the freeze–thaw method (Weige et al., 2006).

### Plant RNA extraction and quantitative RT-PCR

RNA extraction from kiwifruits was carried out using a Quick RNA isolation kit according to the manufacturer’s instructions (#0416–50, Huayueyang, Beijing, China). First-strand cDNA synthesis was performed using 3 μg total RNA and recombinant M-MLV reverse transcriptase (#K1162, Takara, Osaka, Japan). Quantitative PCR (qPCR) was conducted using the Cham QSYBR Color qPCR Master Mix (#Q311, Vazyme, Nanjing, China), and the relative gene expression was analyzed using the 2^−ΔΔCT^ method (Livak & Schmittgen 2001). The Primers used for the qRT-PCR analysis of *AcNAC* are shown in Table S7.

### Construction of AcNAC10 GFP fusion and microscopy imaging of subcellular localization

The correct plasmid was introduced into *Agrobacterium* GV3101 (Weidi, Shanghai, China) using the freeze–thaw method, according to the manufacturer’s instructions. Single colonies were selected and incubated at 28 ℃ for 12–15 h with shaking (220 rpm). The bacterial cultures were collected, resuspended, and adjusted to OD_600_ = 0.6, using MMA solution (10 mM MES (pH 5.6), 2 mM acetosyringone, and 100 mM MgCl_2_). For agroinfiltration, four weeks old *N. benthamiana* leaves were collected and infiltrated with agrobacterial transformants harboring the control plasmid (p1302-35S-GFP) and the AcNAC10 GFP fusion plasmid. Laser confocal microscopy (Olympus, Japan, FV3000) was used to monitor the subcellular localization of AcNAC10 GFP. Images were captured using a 40 × objective with excitation and emission settings of 488 nm for GFP. Images were adjusted using Adobe Illustrator CS5.

### Transcriptional activation of *AcNAC10*

To identify the transcription activation activity of AcNAC10 more accurately, recombinant plasmids pGBKT7-p53 (positive control), pGBKT7-Laminc (negative control), pGBKT7-AcNAC10-N1, pGBKT7-AcNAC10-N2, and pGBKT7-AcNAC10-C were transformed into Y2Hgold cells using the PEG/LiAc method. They were then coated on SD/-Leu/-Trp medium and SD/-Ade/-Trp/-His (+ 4 mg/ml X-α-gal) plates, and cultured at 30 °C for 72–96 h to observe yeast growth status. Primers used to construct the expression vectors are listed in Table S7.

### *Agrobacterium*-mediated transient expression in kiwifruit leaves

Transient expression in kiwifruit leaves was performed as previously described, with slight modifications (Chen et al. 2021). The CDS (coding sequence) of seven NAC genes (Fig. 2C) were cloned and the 35S-AcNACs-GFP fusion expression vector was constructed and transformed into GV3101 *Agrobacterium*. The transformed bacteria were collected and adjusted to OD_600_ = 0.6 using MgCl_2_ solution. Briefly, a two-year-old *A. chinensis* var. *chinensis* Hongyang leaves were punched into discs. The leaf discs were soaked in a 50 ml centrifuge tube containing 30 ml *Agrobacterium* suspension, which was vacuumed at 0.1 Kpa for 15 min. After vacuum filtration, leaf disc surfaces were dried and cultured on 0.5% agar plates at 28 °C for 48 h. Four leaf discs were selected for expression analysis and discs showing overexpression were selected for virulence tests.

### Generation of transgenic plants

The floral dip method was used to generate stable *A. thaliana* (ecotype Columbia 0) lines expressing *AcNAC10* (Clough & Bent 1998). T0 generation seeds were harvested after *Agrobacterium* transformation, sterilized, and seeded on 1/2 Murashige & Skoog (MS) medium containing 75 mg L^−1^ hygromycin. After 7–10 days of incubation at 22 °C (16 h light/8 h dark), plants with normal growth, namely thick green leaves, and the capability to differentiate true leaves, were selected and transplanted into the culture medium (nutrient soil:vermiculite = 3:1) for further culture (22 °C, 16 h light/8 h dark). For identification, DNA was extracted from a small number of leaves when the plants grew three to five true leaves. Tomato lines heterologously expressing *AcNAC10* were generated, as previously described (Chetty et al. 2013). Briefly, Tomato of Ailsa Craig (AC) leaf discs were transformed with the plasmid p1302-*AcNAC10* via *Agrobacterium*-mediated transformation. The plant transformants were regenerated and screened using diagnostic PCR. For positive transformants, the T2 generation plants were used for disease resistance testing.

### Bacterial pathogenesis test

To test the pathogenesis of *Psa*, kiwifruit leaves were inoculated using vacuum infiltration (Liu et al. 2022). The needle puncture method was used to inoculate kiwifruit veins. The GFP-labeled *Psa* bacterial suspension was adjusted to an OD_600_ of 0.1. The needle was then used to puncture the middle part of the vein five times, drawing up 10 μl of the bacterial suspension and dropping it at the puncture sites. After incubation at 16 °C for 7 days, the lengths of the diseased spots were captured under ultraviolet light.

A spray inoculation method was used to examine the pathogenesis of *Pst DC3000* (Zhang et al. 2023). The bacterial suspension was sprayed evenly on both the front and back of the tomato leaves. For *Arabidopsis,* PCR-positive plants were cultured for two to three generations to obtain homozygous transgenic lines. By diluting the final concentration of *Pst DC3000* to OD_600_ = 0.001, four-week-old WT and transgenic *Arabidopsis* leaves were inoculated with needle-free syringes. For inoculation with *Pst DC3000*, activated bacterial cells were suspended in a sterile 10 mM MgCl_2_ solution with an OD_600_ of 0.1. The bacterial suspension was then diluted 20 times and mixed with 0.03% silicone oil by volume before being evenly sprayed on both sides of the leaves of the tomato plants to be treated, ensuring that the amount of bacterial suspension sprayed on each leaf was consistent. After inoculation, the plant environment was strictly controlled (25 °C, relative humidity of approximately 80%, and light cycle of 14 h light/10 h dark).

### Pathogenic bacterial counts

To measure the bacterial load, bacteria-infected leaves were first wiped with 75% ethanol and leaf discs were obtained using a cork borer (diameter = 11 mm). For each sample, three leaf discs were soaked in 1 ml H_2_O and homogenized using a mortar and pestle. A series of dilutions were prepared and 10 μL of each dilution was plated on solid LB supplemented with 50 mg/L kanamycin. The plates were incubated at 28 °C for two days and the number of bacterial colonies was counted. To measure colony-forming units (CFU), the number of colonies formed on plates was multiplied by the dilution factor and normalized to the size of the leaf discs (95 mm^2^). The counts of *Psa* in kiwifruit leaf discs were similar to those in tomato. The silenced plants were inoculated with *Psa*-M228 (OD_600_ = 1 × 10^–5^ cfu/ml) using vacuum infiltration and the inoculated leaf discs were placed on 0.7% agar plates and cultured in a 16 °C incubator. At 5 dpi, the disease incidence in the leaf discs was calculated using Image J software (V.1.53t) (https://imagej.net/), and the colonization of *Psa* in the leaf discs was detected using a method similar to that of tomato.

### Measurement of plant physiological indices

In this study, four-week-old *A. thaliana* and flowering period tomato leaves were inoculated with *Pst DC3000* for 72 h to detect proline, MDA levels, H_2_O_2_ content, and JA content. Plant physiological indexes including proline, MDA, H_2_O_2_, and JA were measured by their corresponding kits, namely proline (Pro) Content Assay Kit (#BC0290, Solarbio, Beijing, China) for proline, Malondialdehyde (MDA) Content Assay Kit (#BC0020, Solarbio, Beijing, China) for MDA, determination of content by using Hydrogen Peroxide (H_2_O_2_) Content Assay Kit (#BC0020, Solarbio, Beijing, China) for H_2_O_2_. To detect JA contents, at 3 dpi, 0.2 g *Psa* infected kiwifruit leaves were collected and frozen at −80 °C until JA analysis. Free JA was analyzed by high-performance liquid chromatography-mass (HPLC–MS/MS) spectrometry.

To observe the production of ROS in genetically modified *Arabidopsis thaliana* after infection with *Pst* DC3000, *A. thaliana* leaves were stained with 3,3’-diaminobenzidine (DAB, 1 mg/mL) or nitroblue tetrazole (NBT, 0.1 mg/mL) at 25 °C for 12 h in the dark. The activity of the antioxidant enzymes was determined using a superoxide dismutase (SOD) kit (#G0101F, Grace, Nanjing, China).

### Yeast one-hybrid assay

The promoter region of *AcNAC10* was selected based on the annotation of Kiwifruit Genome Database and PCR-amplified using primers pHis2-proNAC10-F/R (Table S7). First, four SD/-His/-Trp 90 mm petri dishes were prepared with 3-AT inhibitor self-activation concentrations of 0 mM, 25 mM, 50 mM, and 75 mM. The transformed products were then diluted to the single-clone level (OD < 0.002) and plated. The bait strain, Y187 (pHis2-DNA), was cultured in liquid SD/-Trp medium to prepare competent Y187 (pHis2-DNA) cells. Co-transformation of pGADT7-AcNAC10 and pHis2-proAcLOX3 into Y187 competent cells was performed using the heat-shock method (Froger et al., 2007). Subsequently, all transformed products were plated on SD/-His/-Leu/-Trp + 75 mM 3-AT plates and cultured for 3–5 days.

### β-glucuronidase (GUS) activity assay

The GUS activity was determined as previously described (Jefferson et al. 1987). The effector recombinant vector, p1302-35S-AcNAC10-GFP, was used in the experiment, whereas the reporter recombinant vector, proAcLOX3-GUS, and the negative control, p1302-35S-GFP, were also utilized. *Agrobacterium* harboring effector and reporter expression vectors were mixed in an equal ratio (v/v, 1:1) and co-infiltrated into *N. benthamiana* leaves. Three independent biological replicates were used. After four days, the leaves of each treatment were collected, and GUS activity was measured using a GUS gene quantitative detection kit (#SL7161-100 T, Coolaber, Beijing, China).

An improved method was adopted for the dual-luciferase assay (Alabd et al. 2023). Fusion expression vectors were transformed into *Agrobacterium* GV3101 (pSoup19) using the freeze–thaw method. The proAcNAC10-0800-LUC and p62SK empty vectors were mixed in equal proportion (v/v) and injected into tobacco as the negative control group. Agrobacteria containing the effector plasmid pGreenII 0800-pro*AcNAC10*-LUC and the prey plasmid pGreenII0800-proAcLOX3-LUC were co-infiltrated into *N. benthamiana* and *Agrobacterium* transformants containing the plasmids proAcNAC10-0800-LUC and p62SK were co-infiltrated as controls. The Luciferase (LUC)/Renilla Luciferase (REN) ratio was measured using the Promega chemiluminescence detection system and the Dual-Luciferase Reporter Gene Assay Kit (#11402ES60, Yeasen, Shanghai, China) according to the manufacturer's instructions. Each luciferase assay was performed six times.

### Electrophoretic mobility shift assay (EMSA)

*The AcNAC10* CDS was cloned into the pET15b-GFP-His- tag vector and expressed in *Escherichia coli*. BL21 (DE3). GFP-His-tagged AcNAC10 was purified using a His-tag Protein Purification kit ( #P2226, Beyotime, Shanghai, China). 40-bp forward and reverse primers containing the core sequence of ACGT were synthesized by biotin labeling (5’ and 3’) by Xi'an Qingke Biological Co., Ltd. (Table S7). SDS-PAGE (10%) was used to detect the expression and purification of the AcNAC10 protein (Fig. S14 A). The AcTGA07 protein was purified using the same method as that used for the purification of the AcNAC10 protein (Fig. S14 B). The purified AcNAC10 protein was incubated at 98 °C for 10 min and then slowly cooled to allow for annealing with a double-stranded probe. The purified AcNAC10 protein was then combined with the probe at room temperature for 20 min, and the steps were performed according to the standard EMSA kit from Beyotime (#GS002, Beyotime, Shanghai, China).

### Virus-induced gene silencing (VIGS) of kiwifruit

VIGS was conducted based on a previous report with slight modifications (Chen et al. 2021). The bacterial suspensions of TRV1, TRV2, and TRV1, TRV2-AcNAC10 were mixed in a 1:1 ratio (v/v) for each set, resulting in TRV:00 (TRV1 + TRV2) and TRV:AcNAC10 (TRV1 + TRV2-AcNAC10) infiltration solutions. The mixed *Agrobacterium* solutions were vacuum-infiltrated into the kiwifruit tissue culture seedlings. After 15 days, *AcNAC10* gene expression was measured by qRT-PCR.

## Supplementary Information

Supplementary Material 1

Supplementary Material 2

## Data Availability

All data supporting the fndings of this study are included in the manuscript and its supplementary information.

## Abbreviations

*Psa*: *Pseudomonas syringae* Pv. *actinidiae*
NAC: NAM, ATAF, and CUC
JA: Jasmonic acid
MeJA: Methyl jasmonate
DIECA: Diethyldithiocarbamate
EMSA: Electrophoretic mobility shift assay
Y1H: Yeast one-hybrid
GUS: Beta-glucuronidase
BiFC: Bimolecular fuorescence complementation
VIGS: Virus-induced gene silencing
