# Supplementary material for: AcNAC10, regulated by AcTGA07, enhances kiwifruit resistance to Pseudomonas syringae pv. actinidiae via inhibiting jasmonic acid pathway
Source: Mol Hortic. 2025 Apr 4;5:21. doi: 10.1186/s43897-024-00143-x (PMC11969939; doi:10.1186/s43897-024-00143-x)
Supplement: Supplementary file 1 — Supplementary Material 1 [file 43897_2024_143_MOESM1_ESM.pptx]

## Slide 1
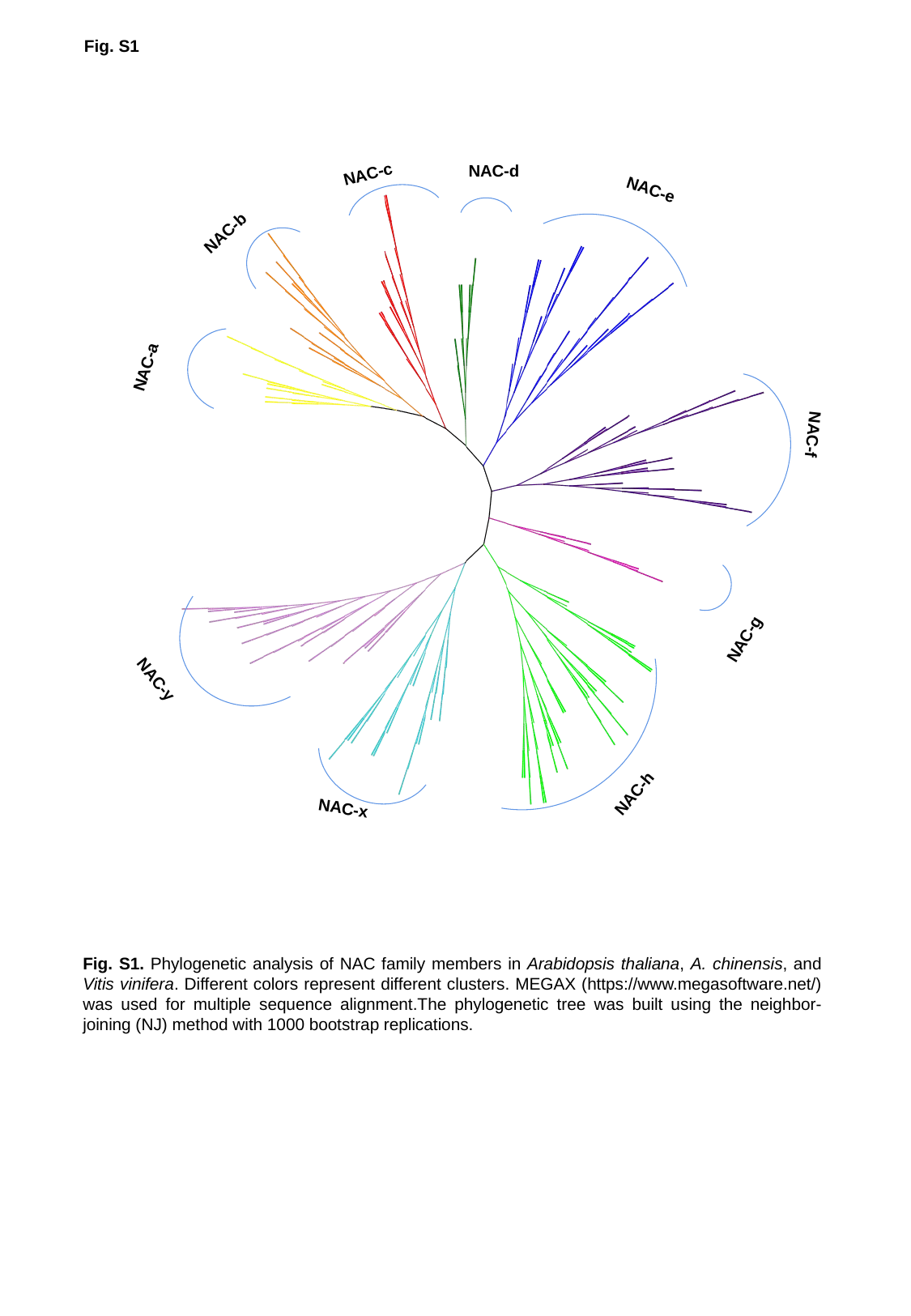

Fig. S1
NAC-c
NAC-d
NAC-e
NAC-b
NAC-a
NAC-f
NAC-g
NAC-y
NAC-h
NAC-x
Fig. S1. Phylogenetic analysis of NAC family members in Arabidopsis thaliana, A. chinensis, and Vitis vinifera. Different colors represent different clusters. MEGAX (https://www.megasoftware.net/) was used for multiple sequence alignment.The phylogenetic tree was built using the neighbor-joining (NJ) method with 1000 bootstrap replications.

## Slide 2
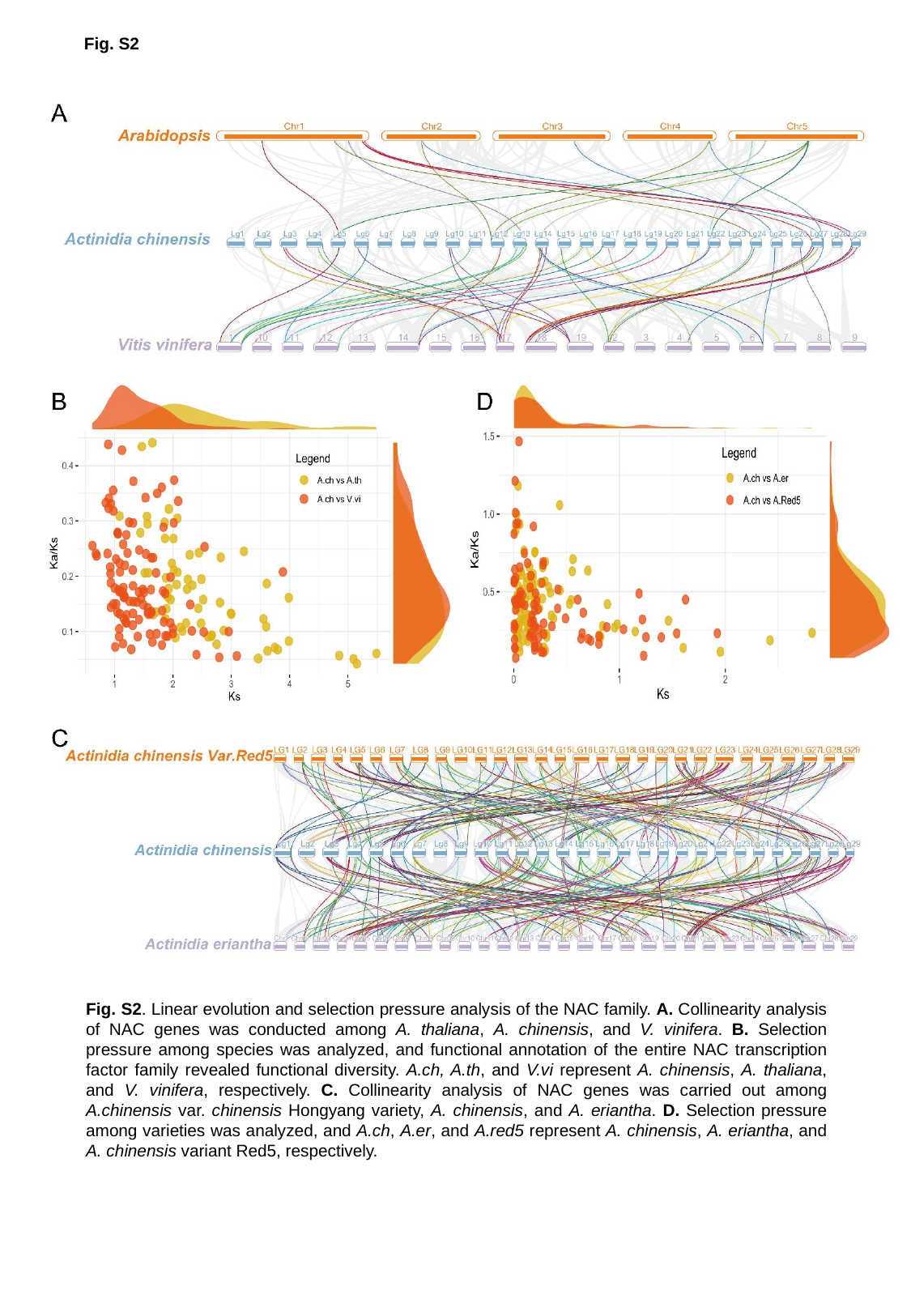

Fig. S2
Fig. S2. Linear evolution and selection pressure analysis of the NAC family. A. Collinearity analysis of NAC genes was conducted among A. thaliana, A. chinensis, and V. vinifera. B. Selection pressure among species was analyzed, and functional annotation of the entire NAC transcription factor family revealed functional diversity. A.ch, A.th, and V.vi represent A. chinensis, A. thaliana, and V. vinifera, respectively. C. Collinearity analysis of NAC genes was carried out among A.chinensis var. chinensis Hongyang variety, A. chinensis, and A. eriantha. D. Selection pressure among varieties was analyzed, and A.ch, A.er, and A.red5 represent A. chinensis, A. eriantha, and A. chinensis variant Red5, respectively.

## Slide 3
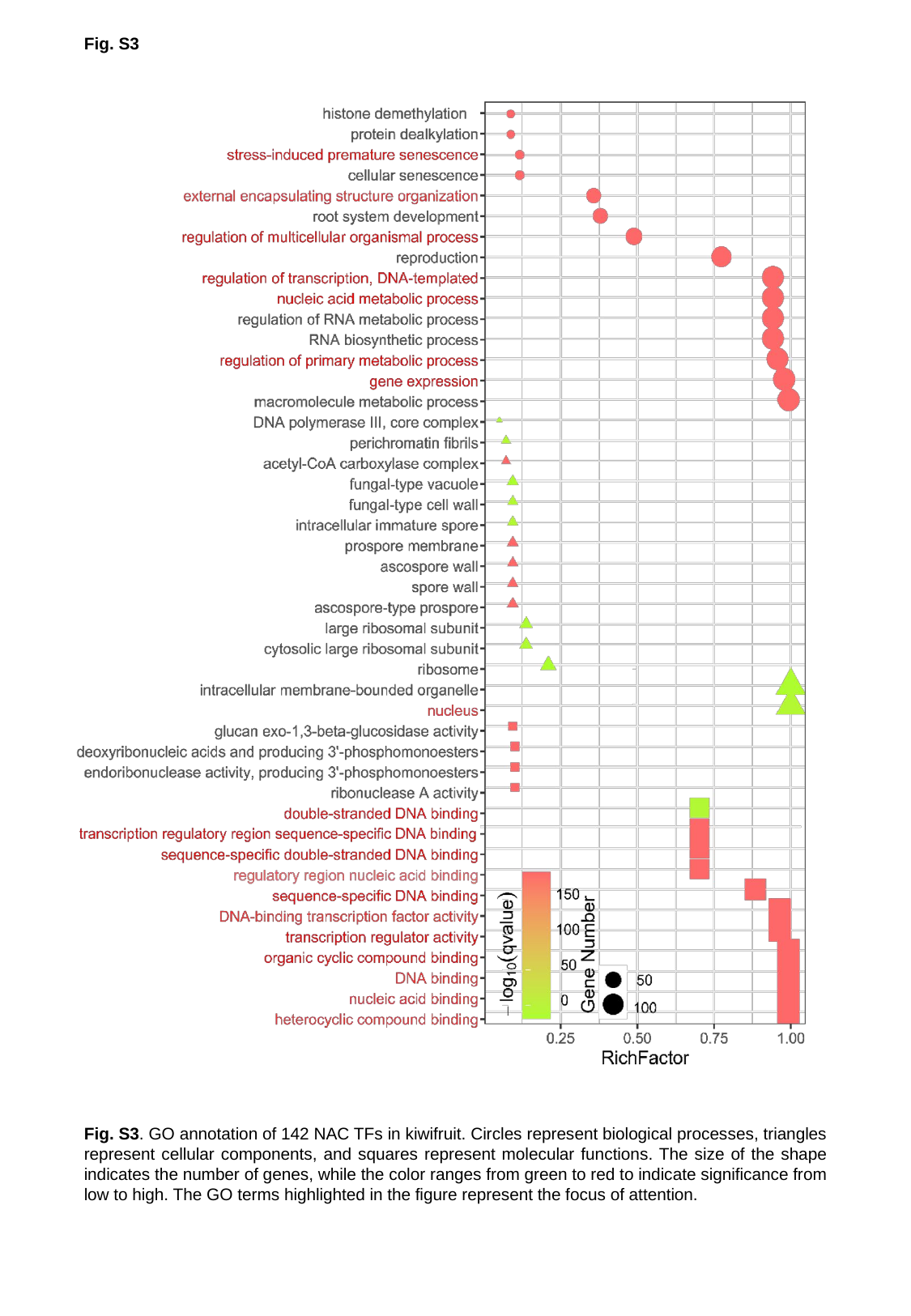

Fig. S3
Fig. S3. GO annotation of 142 NAC TFs in kiwifruit. Circles represent biological processes, triangles represent cellular components, and squares represent molecular functions. The size of the shape indicates the number of genes, while the color ranges from green to red to indicate significance from low to high. The GO terms highlighted in the figure represent the focus of attention.

## Slide 4
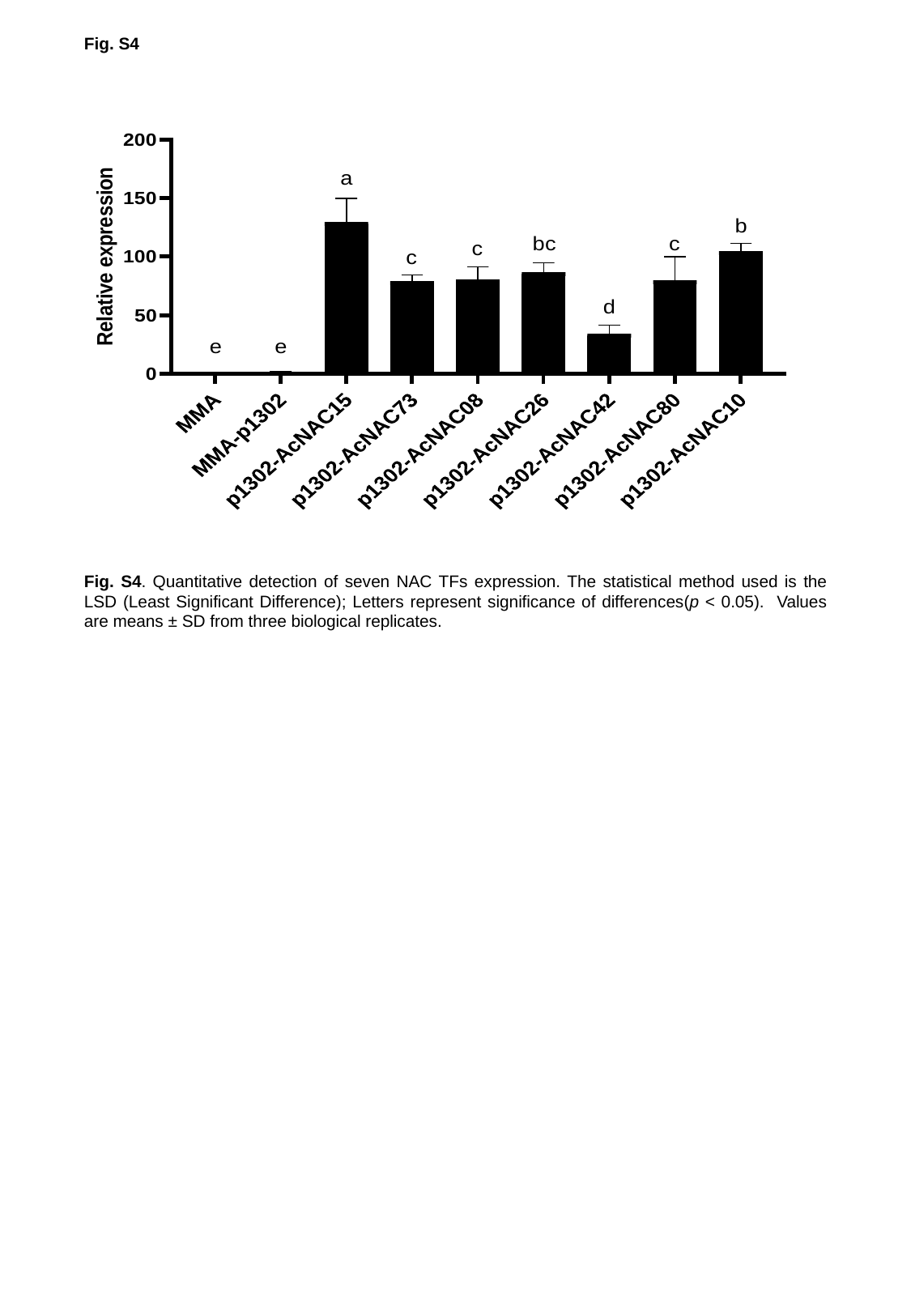

Fig. S4
Fig. S4. Quantitative detection of seven NAC TFs expression. The statistical method used is the LSD (Least Significant Difference); Letters represent significance of differences(p < 0.05). Values are means ± SD from three biological replicates.

## Slide 5
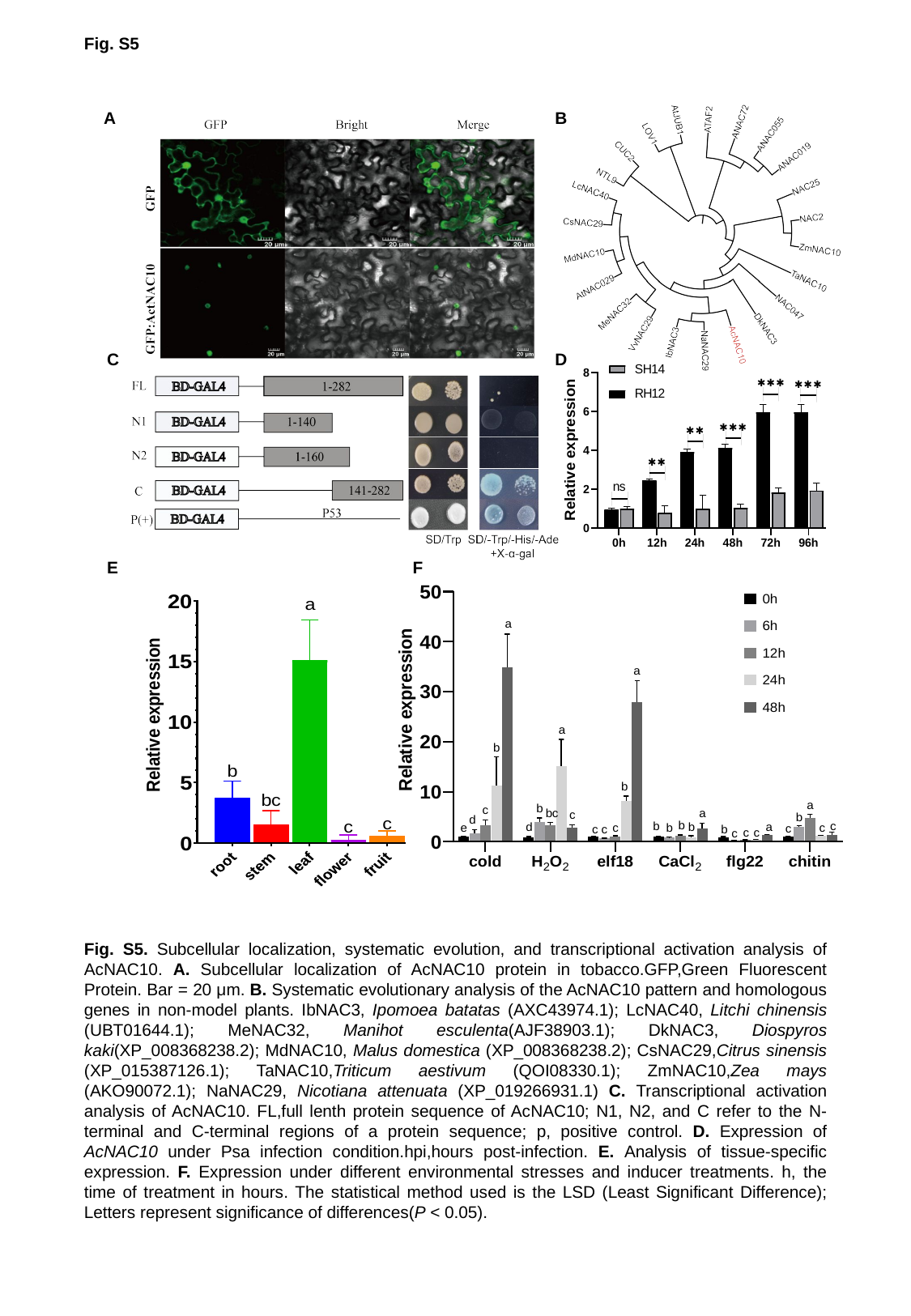

Fig. S5
A
B
C
D
E
F
Fig. S5. Subcellular localization, systematic evolution, and transcriptional activation analysis of AcNAC10. A. Subcellular localization of AcNAC10 protein in tobacco.GFP,Green Fluorescent Protein. Bar = 20 μm. B. Systematic evolutionary analysis of the AcNAC10 pattern and homologous genes in non-model plants. IbNAC3, Ipomoea batatas (AXC43974.1); LcNAC40, Litchi chinensis (UBT01644.1); MeNAC32, Manihot esculenta(AJF38903.1); DkNAC3, Diospyros kaki(XP_008368238.2); MdNAC10, Malus domestica (XP_008368238.2); CsNAC29,Citrus sinensis (XP_015387126.1); TaNAC10,Triticum aestivum (QOI08330.1); ZmNAC10,Zea mays (AKO90072.1); NaNAC29, Nicotiana attenuata (XP_019266931.1) C. Transcriptional activation analysis of AcNAC10. FL,full lenth protein sequence of AcNAC10; N1, N2, and C refer to the N-terminal and C-terminal regions of a protein sequence; p, positive control. D. Expression of AcNAC10 under Psa infection condition.hpi,hours post-infection. E. Analysis of tissue-specific expression. F. Expression under different environmental stresses and inducer treatments. h, the time of treatment in hours. The statistical method used is the LSD (Least Significant Difference); Letters represent significance of differences(P < 0.05).

## Slide 6
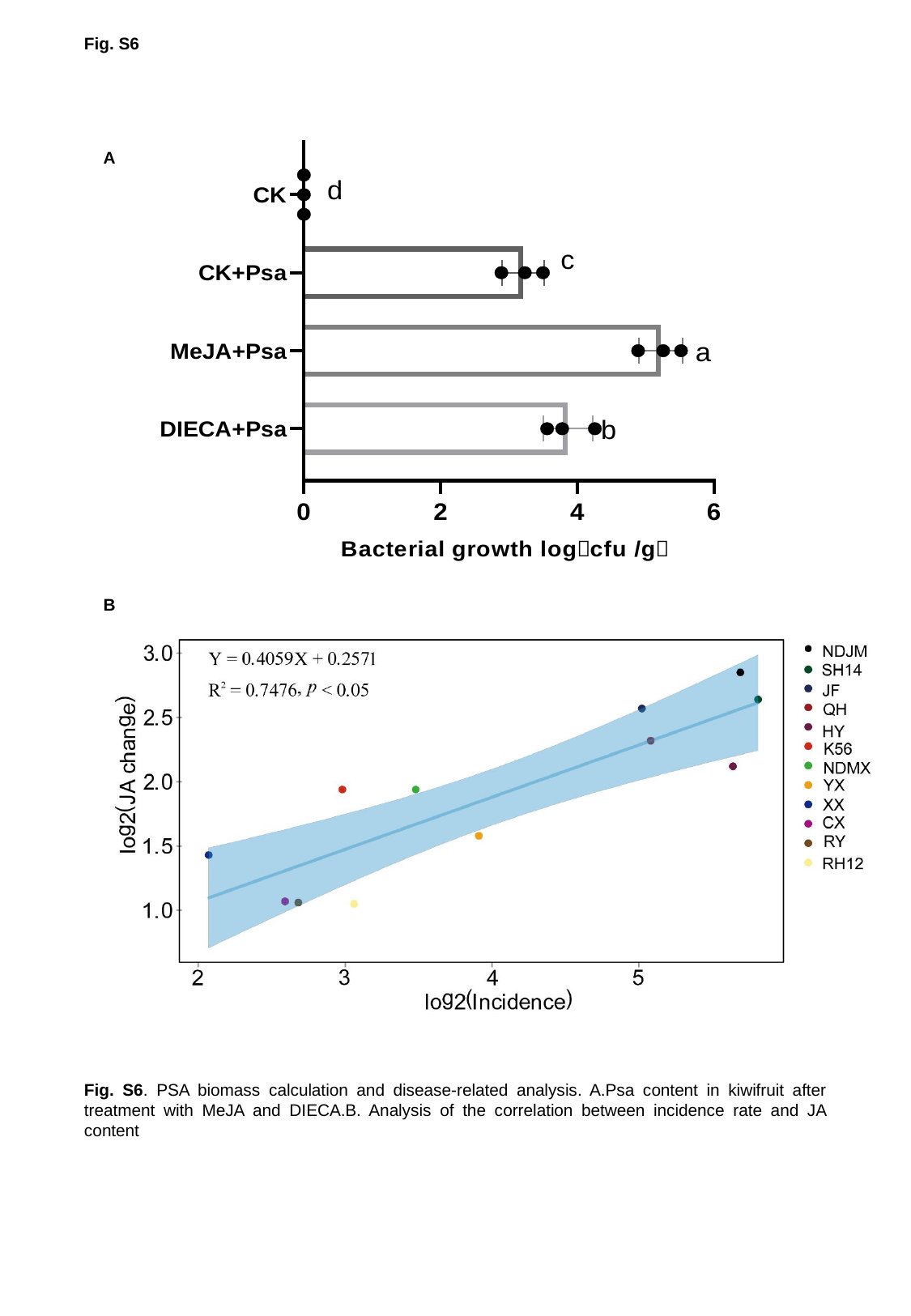

Fig. S6
A
B
Fig. S6. PSA biomass calculation and disease-related analysis. A.Psa content in kiwifruit after treatment with MeJA and DIECA.B. Analysis of the correlation between incidence rate and JA content

## Slide 7
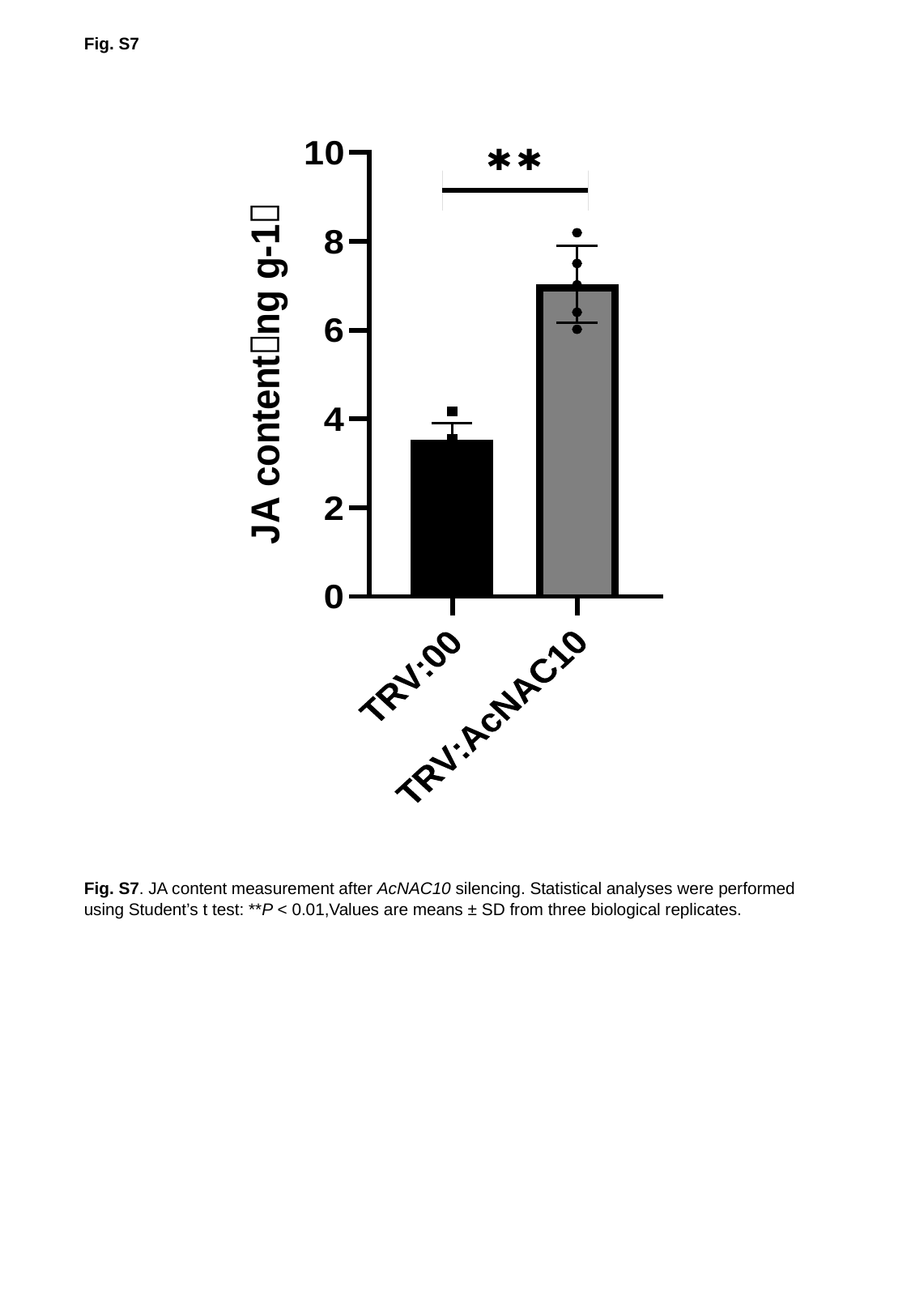

Fig. S7
Fig. S7. JA content measurement after AcNAC10 silencing. Statistical analyses were performed using Student’s t test: **P < 0.01,Values are means ± SD from three biological replicates.

## Slide 8
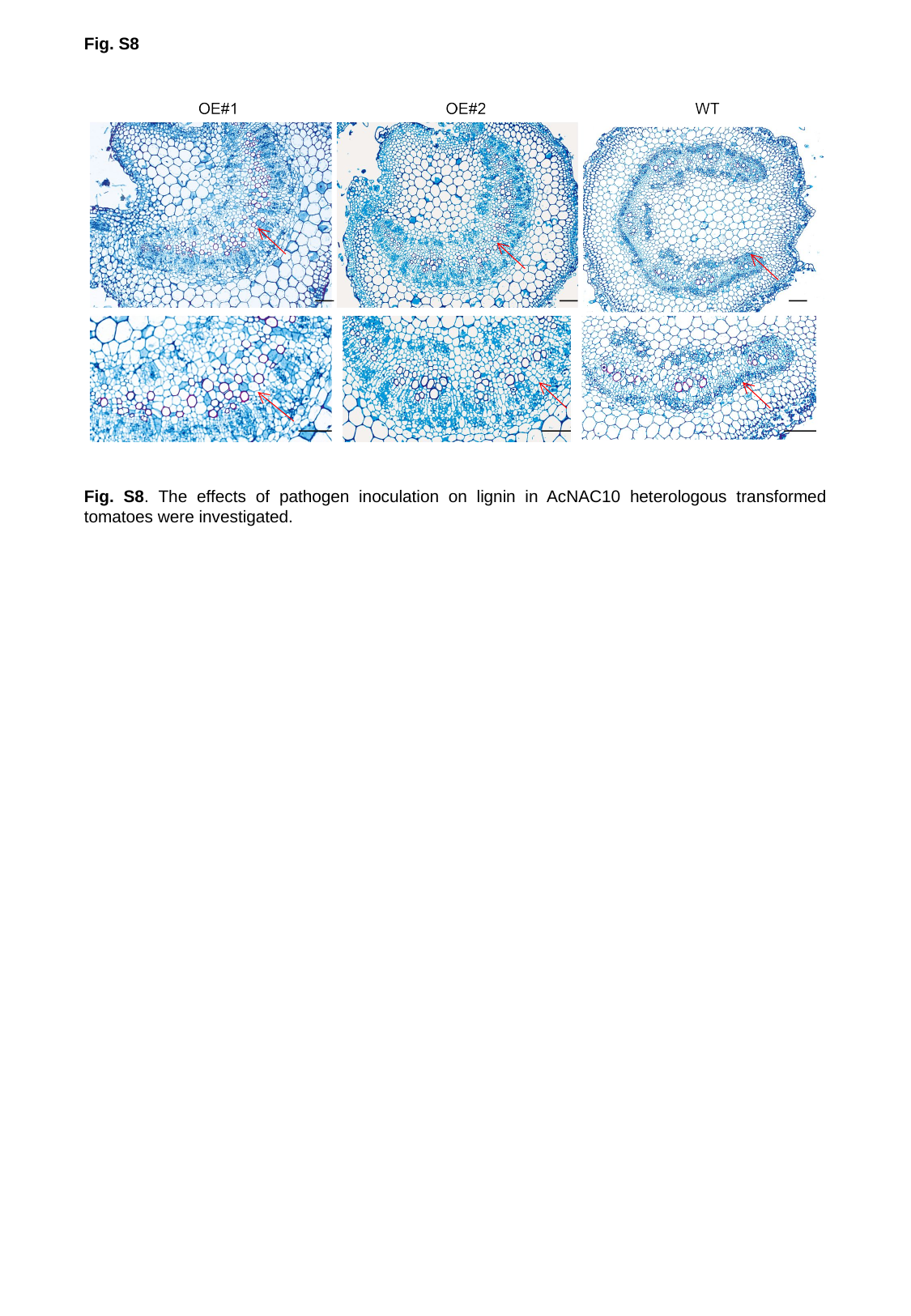

Fig. S8
Fig. S8. The effects of pathogen inoculation on lignin in AcNAC10 heterologous transformed tomatoes were investigated.

## Slide 9
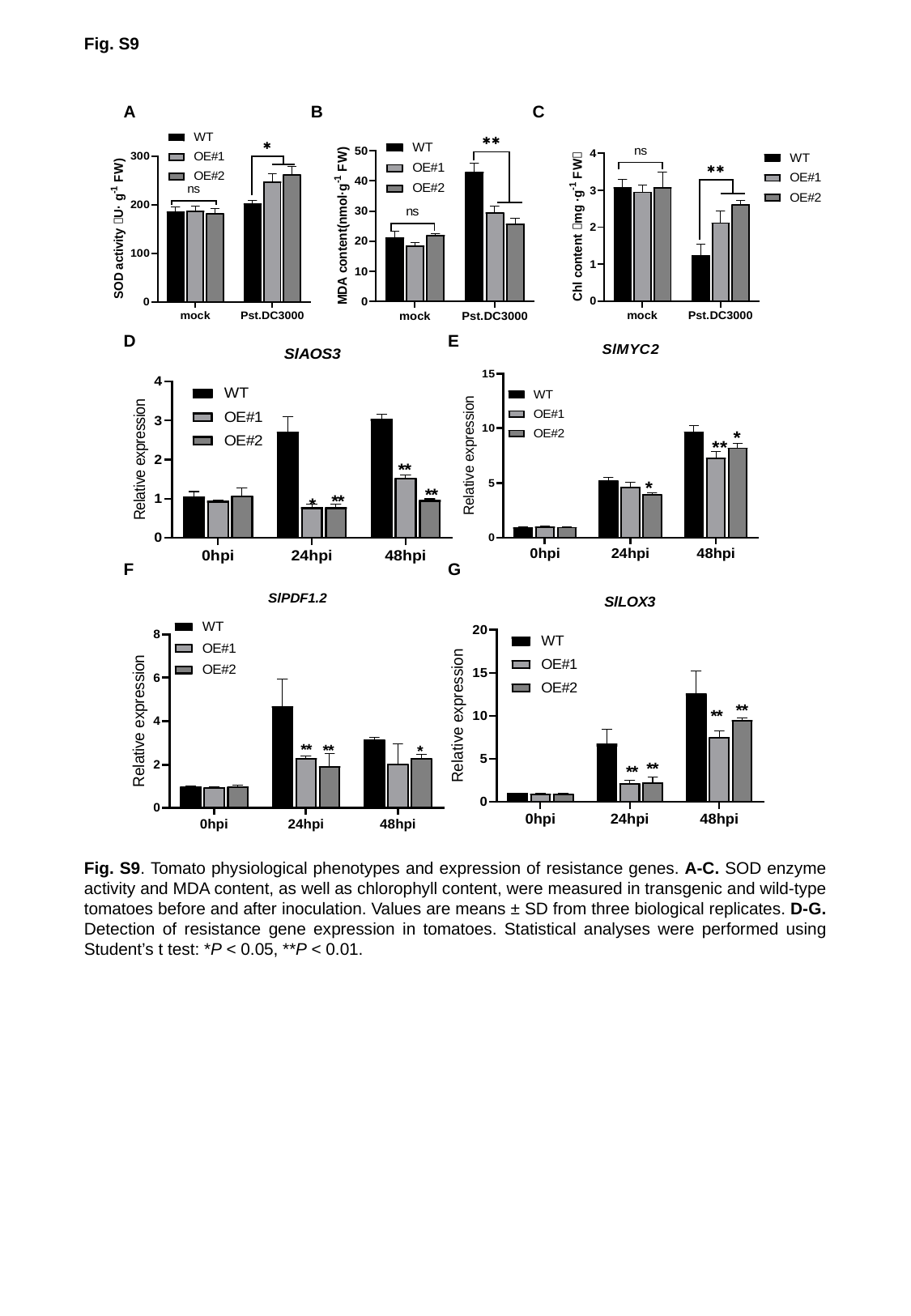

Fig. S9
A
B
C
D
E
F
G
Fig. S9. Tomato physiological phenotypes and expression of resistance genes. A-C. SOD enzyme activity and MDA content, as well as chlorophyll content, were measured in transgenic and wild-type tomatoes before and after inoculation. Values are means ± SD from three biological replicates. D-G. Detection of resistance gene expression in tomatoes. Statistical analyses were performed using Student’s t test: *P < 0.05, **P < 0.01.

## Slide 10
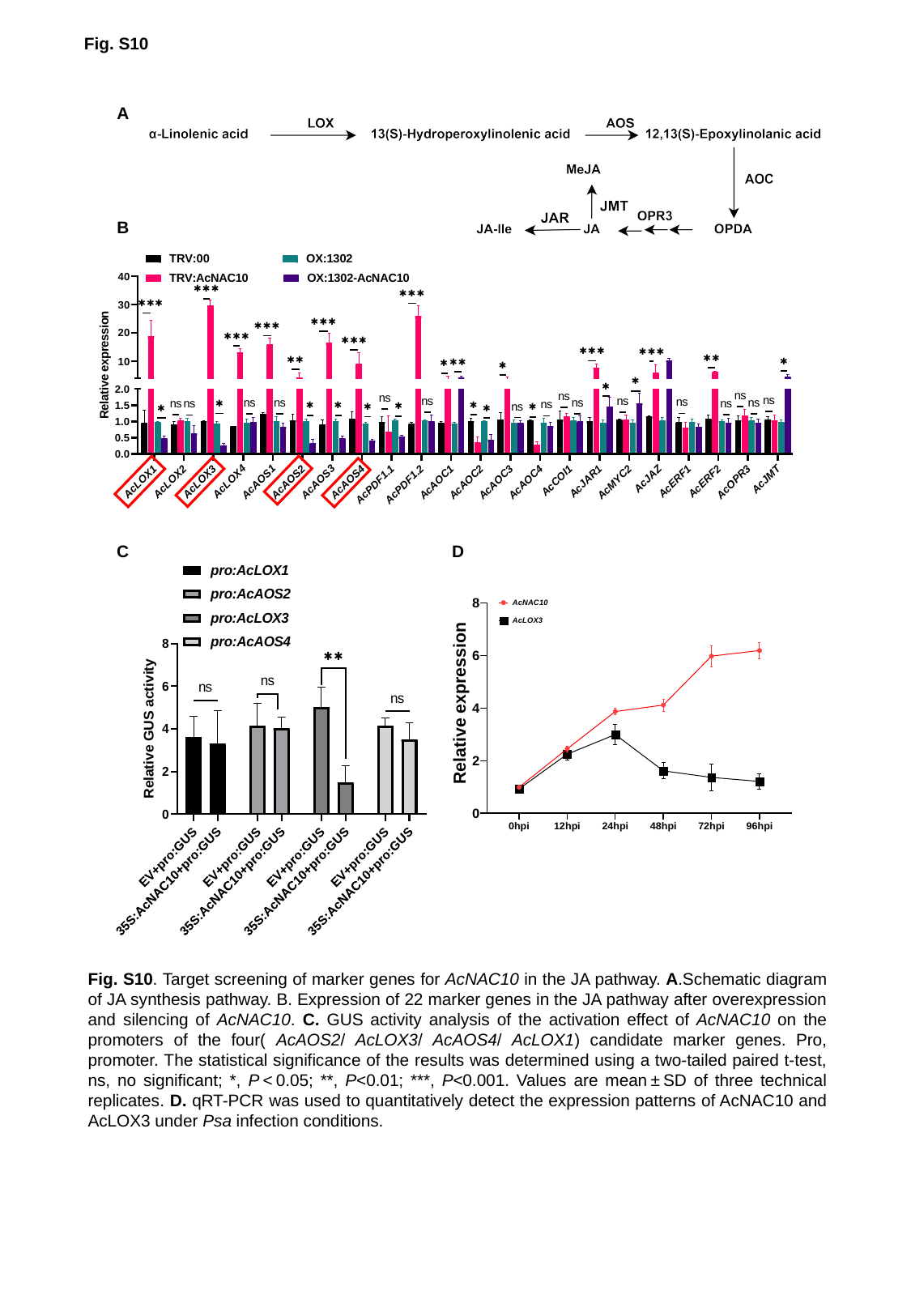

Fig. S10
A
B
C
D
Fig. S10. Target screening of marker genes for AcNAC10 in the JA pathway. A.Schematic diagram of JA synthesis pathway. B. Expression of 22 marker genes in the JA pathway after overexpression and silencing of AcNAC10. C. GUS activity analysis of the activation effect of AcNAC10 on the promoters of the four( AcAOS2/ AcLOX3/ AcAOS4/ AcLOX1) candidate marker genes. Pro, promoter. The statistical significance of the results was determined using a two-tailed paired t-test, ns, no significant; *, P < 0.05; **, P<0.01; ***, P<0.001. Values are mean ± SD of three technical replicates. D. qRT-PCR was used to quantitatively detect the expression patterns of AcNAC10 and AcLOX3 under Psa infection conditions.

## Slide 11
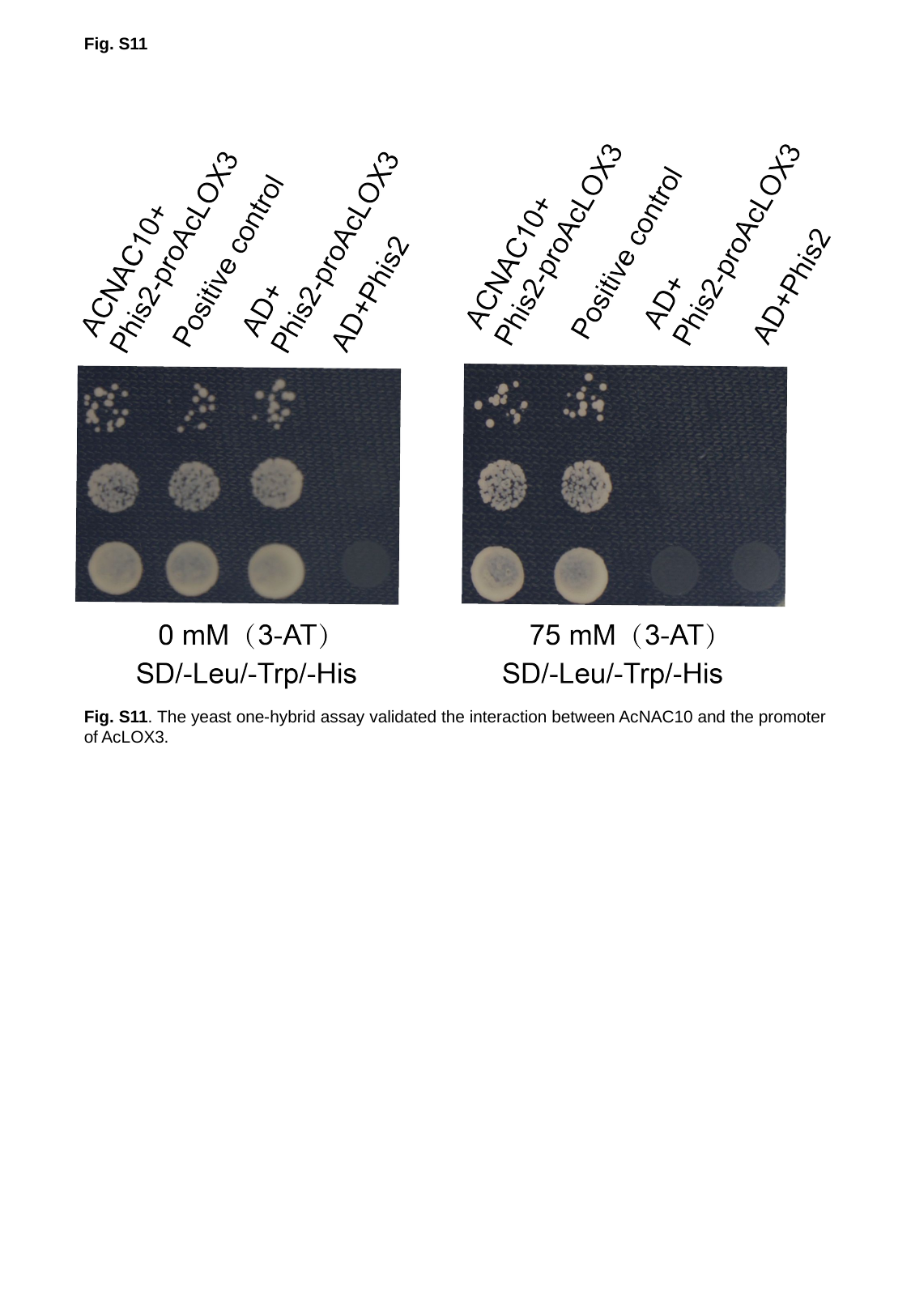

Fig. S11
Fig. S11. The yeast one-hybrid assay validated the interaction between AcNAC10 and the promoter of AcLOX3.

## Slide 12
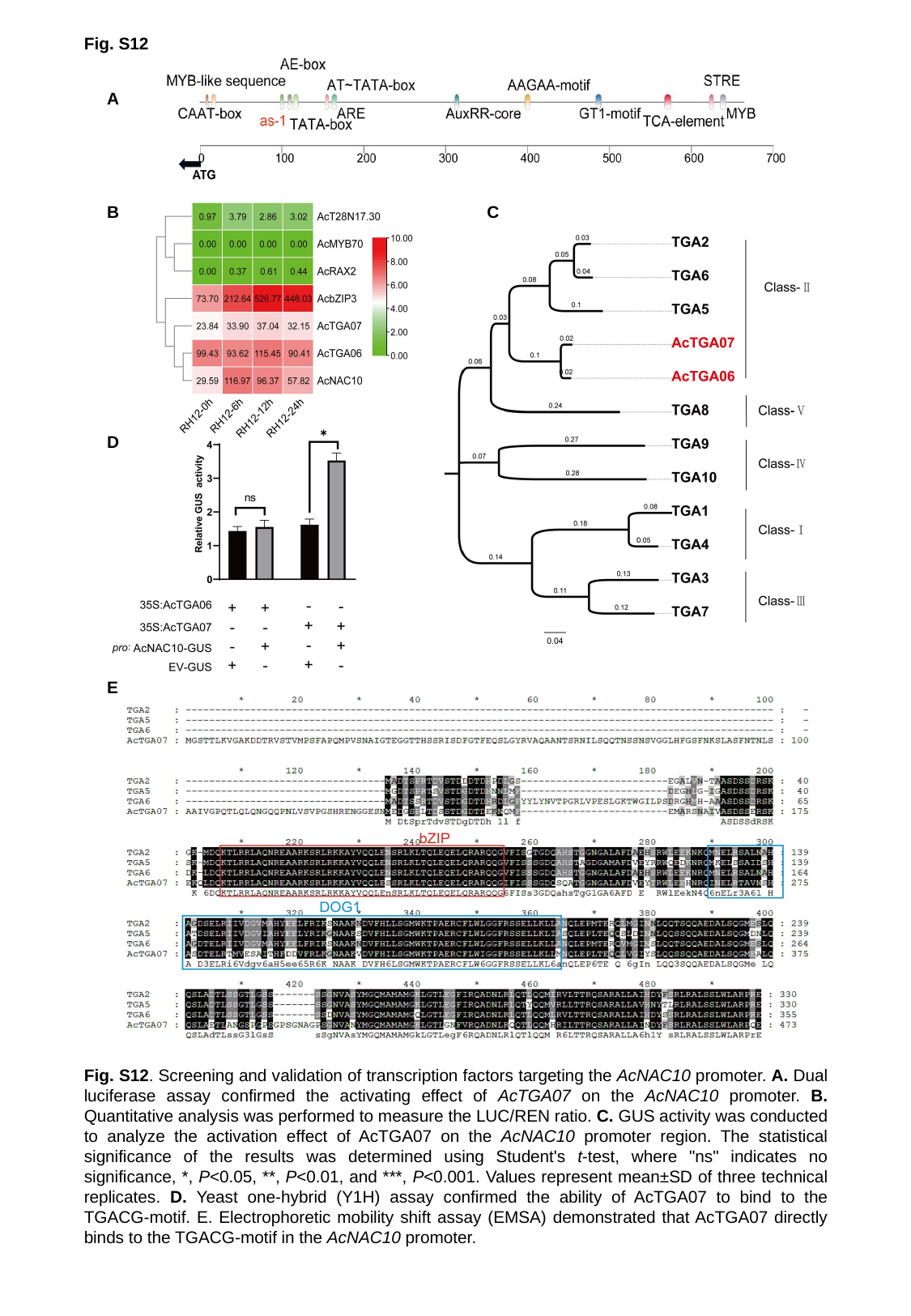

Fig. S12
A
B
C
D
E
Fig. S12. Screening and validation of transcription factors targeting the AcNAC10 promoter. A. Dual luciferase assay confirmed the activating effect of AcTGA07 on the AcNAC10 promoter. B. Quantitative analysis was performed to measure the LUC/REN ratio. C. GUS activity was conducted to analyze the activation effect of AcTGA07 on the AcNAC10 promoter region. The statistical significance of the results was determined using Student's t-test, where "ns" indicates no significance, *, P<0.05, **, P<0.01, and ***, P<0.001. Values represent mean±SD of three technical replicates. D. Yeast one-hybrid (Y1H) assay confirmed the ability of AcTGA07 to bind to the TGACG-motif. E. Electrophoretic mobility shift assay (EMSA) demonstrated that AcTGA07 directly binds to the TGACG-motif in the AcNAC10 promoter.

## Slide 13
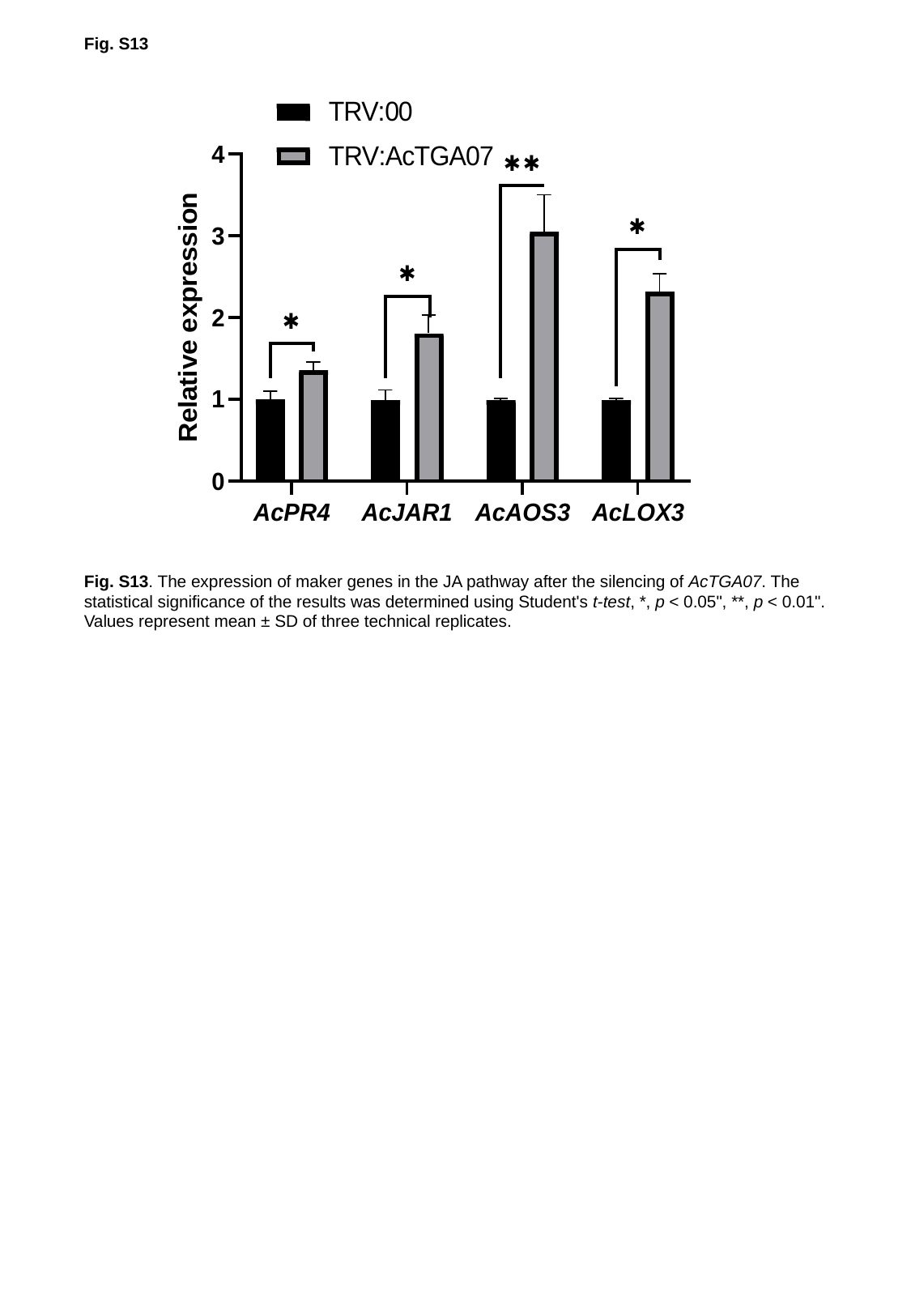

Fig. S13
Fig. S13. The expression of maker genes in the JA pathway after the silencing of AcTGA07. The statistical significance of the results was determined using Student's t-test, *, p < 0.05", **, p < 0.01". Values represent mean ± SD of three technical replicates.

## Slide 14
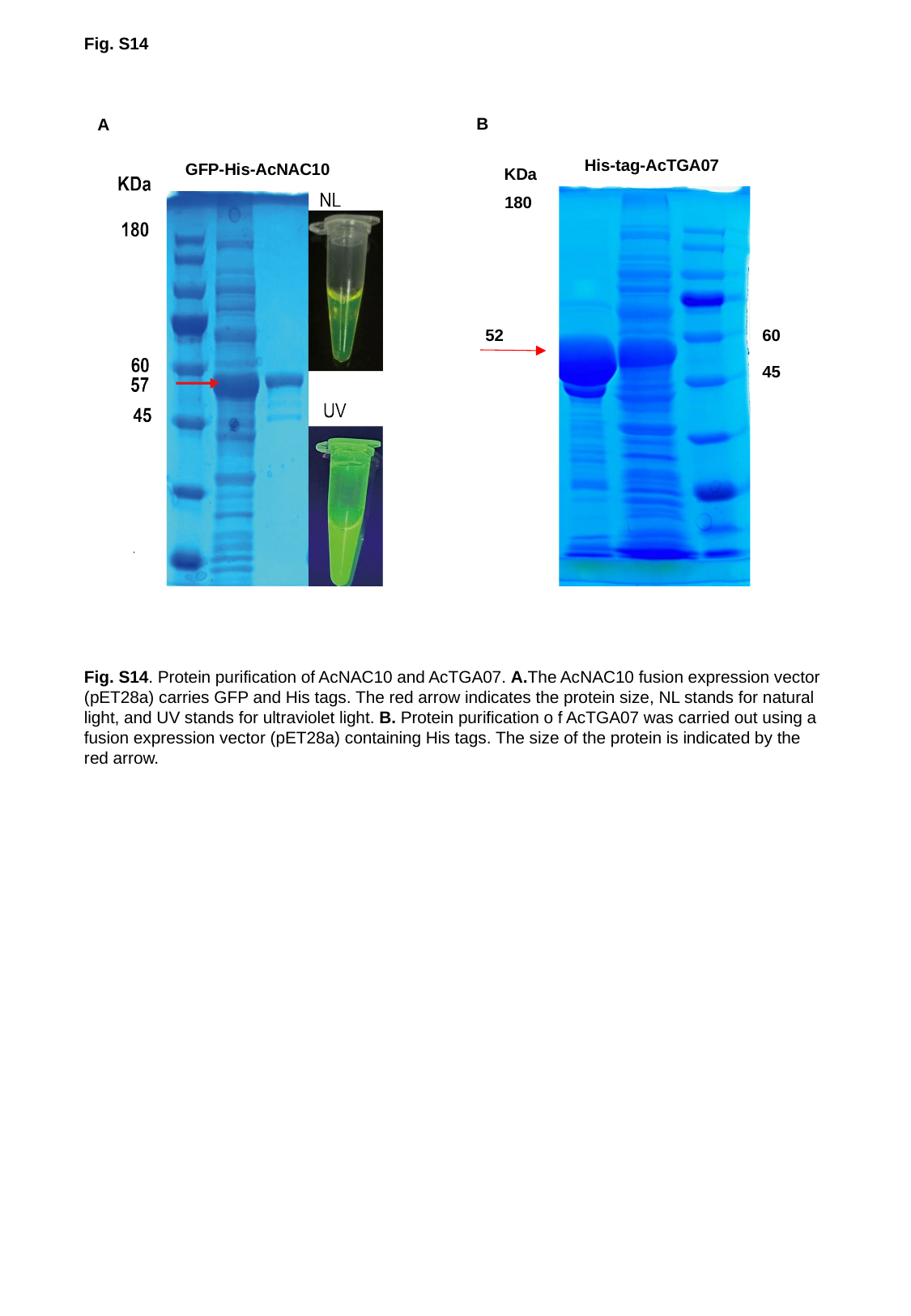

Fig. S14
A
B
His-tag-AcTGA07
KDa
180
52
60
45
GFP-His-AcNAC10
Fig. S14. Protein purification of AcNAC10 and AcTGA07. A.The AcNAC10 fusion expression vector (pET28a) carries GFP and His tags. The red arrow indicates the protein size, NL stands for natural light, and UV stands for ultraviolet light. B. Protein purification o f AcTGA07 was carried out using a fusion expression vector (pET28a) containing His tags. The size of the protein is indicated by the red arrow.
