# Supplementary material for: AcNAC10, regulated by AcTGA07, enhances kiwifruit resistance to Pseudomonas syringae pv. actinidiae via inhibiting jasmonic acid pathway
Source: Mol Hortic. 2025 Apr 4;5:21. doi: 10.1186/s43897-024-00143-x (PMC11969939; doi:10.1186/s43897-024-00143-x)
Supplement: Supplementary file 2 — Supplementary Material 2 [file 43897_2024_143_MOESM2_ESM.docx]

**Supplemental Tables :**

**TableS1. Phylogenetic tree statistics of *Vitis vinifera*, *Arabidopsis thaliana* and *Actinidia chinensis***

**TableS2. Statistical analysis of similarity between *AcNAC10* and homologous genes in other species.**

| GeneName | Similarity (%) |
| --- | --- |
| IbNAC3 | 77.7 |
| LcNAC40 | 78.55 |
| MeNAC32 | 81.05 |
| DkNAC3 | 72.28 |
| VvNAC29 | 81.4 |
| MdNAC10 | 75.69 |
| CsNAC29 | 73.31 |
| AcNAC10 | 100 |
| TaNAC10 | 48.27 |
| ZmNAC10 | 41.16 |
| NaNAC29 | 77.66 |
| AtNAC029 | 61.17 |
| AtNAC25 | 51.69 |
| AtNAC2 | 44.36 |
| AtNAC047 | 51.21 |
| AtCUC2 | 41.81 |
| AtATAF2 | 50.47 |
| AtNAC019 | 52.96 |
| AtNAC055 | 52.55 |

**TableS3. Prediction of *cis*-acting elements in the AcNAC10 promoter region.**

**TableS4. The potential interacting candidate targets of AcNAC10 identified through Y1H screening.**

| **Categories** | **ID** | **GeneName** | **Descriptions** |
| --- | --- | --- | --- |
| Transcription factors | Actinidia38164.t1 | AcTGA06 | Transcription factor like |
|  | Actinidia17695.t1 | AcTGA07 | Transcription factor like |
|  | Actinidia00411.t1 | AcMYB70 | MYB transcription factor |
|  | Actinidia00046.t1 | AcT28N17.30 | Zinc finger CCCH domain-containing protein |
|  | Actinidia00544.t1 | AcbZIP3 | BZIP transcription factor |
|  | Actinidia01210.t1 | AcRAX2 | MYB transcription factor |
| Other proteins | Actinidia16231.t5 | AcTBP1 | TATA-box-binding protein 1 |
|  | Actinidia39299.t1 | AcSYP124 | Syntaxin, putative |
|  | Actinidia18783.t1 | AcRABA1F | Ras-related protein like |
|  | Actinidia35230.t1 | AcRABA1F | Ras-related protein like |
|  | Actinidia39278.t1 | AcAPUM21 | Pumilio 12 like |
|  | Actinidia39323.t1 | AcF20O9.80 | Protein phosphatase 2c, putative |
|  | Actinidia05309.t1 | AcPLL4 | Protein phosphatase 2c, putative |
|  | Actinidia28046.t1 | AcDHS2 | Phospho-2-dehydro-3-deoxyheptonate aldolase |
|  | Actinidia29257.t1 | AcDHS2 | Phospho-2-dehydro-3-deoxyheptonate aldolase |
|  | Actinidia39243.t3 | AcF24G16.250 | Nucleic acid-binding, OB-fold-like protein. |
|  | Actinidia37074.t1 | AcDCP1 | mRNA-decapping enzyme-like protein |
|  | Actinidia13206.t1 | AcF7H2.14 | IQ domain-containing protein |
|  | Actinidia00536.t1 | AcF16F14.15 | F-box protein |
|  | Actinidia36006.t1 | AcF16F14.15 | F-box protein |
|  | Actinidia07886.t1 | AcLHCB2.4 | Chlorophyll a-b binding protein, chloroplastic |
|  | Actinidia37251.t1 | AcRPL35B | 60S ribosomal protein L35 |
|  | Actinidia37233.t1 | AcRPS15AD | 40S ribosomal protein |
| Enzymes | Actinidia37086.t1 | AcSKD1 | ATP-dependent zinc metalloprotease FtsH 1 |
|  | Actinidia13447.t1 | CIPK23 | CBL-interacting serine/threonine-protein kinase 23 |
|  | Actinidia03346.t2 | CIPK23 | CBL-interacting serine/threonine-protein kinase 23 |
|  | Actinidia27509.t1 | CIPK23 | CBL-interacting serine/threonine-protein kinase 23 |
|  | Actinidia36689.t1 | AcT31B5.60 | Repressor of RNA polymerase III transcription |
|  | Actinidia37053.t1 | AcLRK10L-1.1 | Receptor-like protein kinase |
|  | Actinidia12413.t1 | AcF23M19.5 | Receptor-like kinase |
|  | Actinidia12560.t1 | AcCRPK1 | Receptor-like kinase |
|  | Actinidia39248.t1 | AcA0A1I9LP65 | Polygalacturonase |
|  | Actinidia39250.t1 | AcA0A1I9LP65 | Polygalacturonase |
|  | Actinidia03142.t1 | AcMXC20.6 | NADH dehydrogenase 1 alpha subcomplex subunit 5 |
|  | Actinidia03632.t1 | AcAMC4 | Metacaspase |
|  | Actinidia15394.t1 | AcLDL3 | Lysine-specific histone demethylase like |
|  | Actinidia37091.t1 | AcHDA9 | Histone deacetylase |
|  | Actinidia15327.t1 | AcF22H5.9 | Erythronate-4-phosphate dehydrogenase family protein |
|  | Actinidia22548.t1 | AcF18O14.16 | Erythronate-4-phosphate dehydrogenase family protein |
| Uncharacterized proteins | Actinidia39267.t1 | AcF12F1.10 | Protein of unknown function (DUF506) |
|  | Actinidia19027.t1 | AcF6A14.17 | DUF962 domain-containing protein |
|  | Actinidia35007.t1 | AcF6A14.17 | DUF962 domain-containing protein |
|  | Actinidia39535.t1 | AcOBAP2A | DUF1264 domain-containing protein |

**TableS5. Statistical analysis of similarity(%) between AcTGA07 and homologous proteins in *Arabidopsis thaliana*.**

| **Gene Name** | TGA2 | TGA5 | TGA6 | AcTGA07 |
| --- | --- | --- | --- | --- |
| TGA2 | 100 | 94.58 | 92.57 | 64.06 |
| TGA5 | 94.58 | 100 | 90.36 | 62.85 |
| TGA6 | 92.57 | 90.36 | 100 | 59.24 |
| AcTGA07 | 64.06 | 62.85 | 59.24 | 100 |

**TableS6. Promoter sequences of *AcNAC10* and *AcTGA07***

> ***AcLOX3* promoter (1-836bp)**

5’-CAGACTCTTACAGCTTGCTGCTCGCCGTGTCGAGGTGGCAATCCCTTCCGGGATCTTATGCATACTGGTGCCGAGAGTGAGCAGAATGGCTGTGCGCTTGATCGATTTTGGCAACGGCTGCACCGATTTTTGCATTAAATCCTGCGGATGACCATGTGGCAACATGCGGACCAGGCCAAAATAGCCAAGCAGACCGAAGATAAACATTCCATAACCCAACACAGGCGACATTCCTTCAGCTGCTAGTGCGGCAGGAAGCATTTCCATTAATGAGATGAGCAACATGATCCCCGCCGCAAAACCTAACGAAAACGCCAGTAAGCGGTTCGAGGGTTTTTGCCCGATAACGCCGAAAAACGCGCCAATAAACGTGGCTGCCCCCGCCAGTATGGTCATAATGAGAGGTACTGACATCGACAACTCCTTATAAATCTTCACCTTCAGGCAGATCGTCAAAATAACTGATGATCATCATCATTGTTATCCGGGTTCTTACTCCGCCGAACAAGCGTATTGTGAGGATATCAAAATGACACCTTTAGTTAAGGATATCATCATGTCTTCAACACGTATGCCAGCATTGTTTTTTAGGGTCAGGGTAGTCCGATGAACGTGCTGGAAGATAATTTGTATACCCGCAGCTGGCAGAAGTTGGGGATGACATTGCCACGCCCGCAAGCGATTGTGGTGGCTGCGGCTCACTGGGTTTACCCGTGTGAACATGAGTGACCGCGATGGAGACGCCGCCCACGATTTTGCTGTGGGGGCCCCCAAACAAAATTAAAACACAGGTTCCCTCTCGGGTTTCTCCCGGGGGAAAAACCATGTGGCCCCAAATG-3’

**>*AcNAC10* promoter (1-883bp)**

5’-CTGAGCTTTTCTTTCCCACCATTGTTTCTATAGTAATATATATCAAATAAATTCAAAAATTTAAACCTGAAAAGAAAAAGACCAAAATCAAAAGAGGGTAACAAATATTGGTTCCAAGTCCACCAAAAAGAAAGACGATTATTTTTTTTTTGCTTAAAAAAACAATATTGGTTTGGTTAATAGGGGGGGATGGGGGGAGGGGGAAAAAAAAAGGTTTAAAAAGGAAAAAAAAAAAGGAGGGGGTTTGAAAGGTTGGAAGGGAAAGAAGTGGCCCTTTATTGGGGGGGGTTTTTTGGCCGGGTGGGGGGAATACCTCCCCTTGGATGGGTGGAACTTGGCTTCCCCCACCCCCGATGAACATTGGATGGCCTACTCCCAGGGGGAAGGAAAAAGGTTTACTTCCTTCCTAAAAATGGCCACCCATATTAAGGGGGTATCATTTCATTTTCCTATTAGAATATTTTGGGTTCACACCCTGGCCAGAAGGACCATTAAGGGTCCATATTCCCCTCCCCCCATTATTTCACACTAATCCATTTTTGGTATAAATTAAACCATTCCTGACGTTTTGGTCCTAAAAATCTGCTCTACACCCAATGCAGACTCAGTATGGATTGTGTCAATGCTCTTTGAGATCTAAATTACATGTGGATCAATATATCATATG-3’

Note:The red sequence indicates the possible NACRS/as-1 sequence bound by AcNAC10/ AcTGA07 protein.The "ATG" in blue font represents the transcription start site

**TableS7. All primers used in this study**
